# Revitalizing nanoscale solid–solid conversion enables ultrastable aqueous batteries

**DOI:** 10.1093/nsr/nwag010

**Published:** 2026-01-13

**Authors:** Zhixin Sun, Mei Han, Yuchun Liu, Hang Wang, Xingwu Zhai, Liang Wu, Zhuohui Zhang, Jian Zhi, Pu Chen, Min Zhou

**Affiliations:** Hefei National Laboratory for Physical Sciences at the Microscale, School of Chemistry and Materials Science, University of Science and Technology of China, Hefei 230026, China; State Key Laboratory of High Performance Ceramics, Shanghai Institute of Ceramics, Chinese Academy of Sciences, Shanghai 200050, China; Department of Chemical Engineering and Waterloo Institute of Nanotechnology, University of Waterloo, Waterloo N2L 3G1, Canada; Hefei National Laboratory for Physical Sciences at the Microscale, School of Chemistry and Materials Science, University of Science and Technology of China, Hefei 230026, China; Hefei National Laboratory for Physical Sciences at the Microscale, School of Chemistry and Materials Science, University of Science and Technology of China, Hefei 230026, China; Hefei National Laboratory for Physical Sciences at the Microscale, School of Chemistry and Materials Science, University of Science and Technology of China, Hefei 230026, China; Hefei National Laboratory for Physical Sciences at the Microscale, School of Chemistry and Materials Science, University of Science and Technology of China, Hefei 230026, China; Hefei National Laboratory for Physical Sciences at the Microscale, School of Chemistry and Materials Science, University of Science and Technology of China, Hefei 230026, China; State Key Laboratory of High Performance Ceramics, Shanghai Institute of Ceramics, Chinese Academy of Sciences, Shanghai 200050, China; Department of Chemical Engineering and Waterloo Institute of Nanotechnology, University of Waterloo, Waterloo N2L 3G1, Canada; School of Chemical and Biomolecular Engineering, College of Engineering, Eastern Institute of Technology, Ningbo 315200, China; Hefei National Laboratory for Physical Sciences at the Microscale, School of Chemistry and Materials Science, University of Science and Technology of China, Hefei 230026, China

**Keywords:** size-revitalizing layer, solid–solid conversion, size-controlled products, high mass loading, aqueous zinc–manganese batteries

## Abstract

Aqueous zinc–manganese (Zn–Mn) batteries driven by deposition–dissolution reactions hold significant promise for large-scale grid energy storage. However, their lifetimes are limited by incomplete solid–solid conversion, driven by irreversible pathways forming large-sized solid products—a critical yet overlooked size-dependent challenge. Here, we construct a size-revitalizing layer (SRL) on MnO_2_ to regulate the interfacial microenvironment via sustained Mn release and strong interaction with solid products. The SRL governs growth kinetics, stabilizing nanoscale products formation. Such small solid products shorten ion-diffusion paths and reduce concentration polarization, enabling a reversible Mn (Ⅱ, *l*)–Mn (Ⅳ, *s*) pathway instead of an irreversible Mn (II, *l*)–Mn (III, *s*) route. Systematic screening identifies Bi_2_O_3_ as the optimal modifier based on its strong p–s (M–Zn) orbital interaction and lattice compatibility, which reduce solid product size from >10 μm to the nanoscale, achieving unparalleled stability and resolving irreversible capacity degradation. With a high-mass-loading cathode (9 mg cm^−2^), coin cells achieve >1000 cycles at 2 C and scaled iron-plate cells (16 mg cm^−2^, 26.3 mAh) operate stably for 110 days. This size-controlled solid–solid conversion strategy exhibits broad applicability to diverse electrode materials, highlighting its potential for widespread adoption in advanced energy-storage systems.

## Teaser

A new coating strategy enables ultra-stable zinc-manganese batteries by controlling the growth of key components at the nanoscale, offering a practical path for large-scale renewable energy storage.

## INTRODUCTION

**Figure 1.**
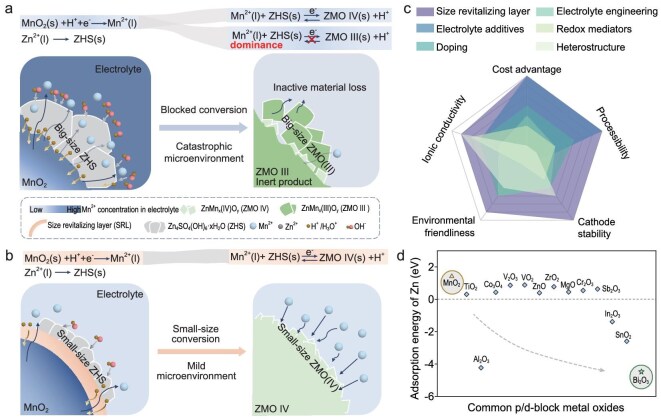
Regulation effect and design principles of the size-revitalizing layer. (a) Schematic diagram of the failure process of the MnO_2_ cathode. (b) Schematic diagram of the SRL effect on regulating the size of the solid–solid conversion. (c) Comparison of the properties of different modification strategies. (d) Adsorption energy of Zn by different oxides.

Reversible solid–solid phase transition with high theoretical capacity has emerged as one of the dominant paradigms for electrochemical energy storage, such as typical conversion-type ion batteries, metal–sulfur batteries and metal–gas batteries [1–5]. In this context, the deposition–dissolution mechanism in mildly acidic aqueous Zn–Mn batteries has recently been recognized as a prototypical solid–solid transition process [6,7], in which the ‘deposition’ and ‘dissolution’ of Mn species are fundamentally governed by coherent solid–solid transition between zinc hydroxyl sulfate (Zn_4_SO_4_(OH)_6_·*x*H_2_O, ZHS) and zinc Woodruffite (ZnMn*_x_*O*_y_*, ZMO) [8,9]. While the multivalent nature of Mn enables multi-electron redox reactions that enhance theoretical energy density [10–12], the slow kinetics of solid-phase reactions lead to significant capacity decay during long-term operation, which is derived from the irreversible Mn (II, *l*)-to-Mn (III, *s*) reaction pathway and the accumulation of electrochemically inactive Mn species (dead Mn, Fig. 1a) [13,14].

Recent breakthroughs involve electrolyte additives, redox mediators and colloid electrolytes [13,15–18]. However, electrolyte engineering suffers from decomposition and loss in long-term cycling [19]; redox mediators suffer from parasitic reactions with Zn metal [20], leading to limited lifetime of batteries that is not desirable for practical applications. Therefore, achieving a long operation life under industrially relevant rate (<0.5 C) and mass loading (>10 mg cm^−2^) is still far from reality [21,22].

The size reduction of solid phases offers a promising avenue to enhance the reaction efficiency between ZHS and ZMO. Smaller solid phases increase reaction interfaces and diffusion cross sections, providing more reaction sites for subsequent deposition or growth [23]. They shorten the ion-diffusion paths and reduce concentration polarization [24], thereby facilitating optimal reaction equilibrium and inducing a reversible Mn (Ⅱ, *l*)–Mn (Ⅳ, *s*) pathway (Fig. 1b). However, the initial rapid dissolution of MnO_2_ and accompanied fluctuations in the concentration of H^+^ ions make the size-controlled transition between solid-phase ZHS and ZMO a formidable challenge [25]. Here, we report that constructing a size-revitalizing layer (SRL) on the MnO_2_ surface could achieve a remarkable reduction in solid product size from tens of μm to the nanometer scale, addressing the critical challenges of irreversible capacity degradation in the Zn–Mn aqueous battery. When we refer to ‘solid–solid conversion’, we are primarily emphasizing that the bulk states of the active materials before and after the reaction are both solid. For the effective size modulation of solid–solid conversion and to form a robust and ionic selective SRL on MnO_2_ particles, the SRL must fulfill three criteria: (ⅰ) have strong binding to the MnO_2_ particle to ensure structural integrity and prevent SRL detachment during charge/discharge cycles, (ii) have strong adsorption of Zn to promote uniform nucleation and deposition growth of ZHS and ZMO, minimizing side reactions during the solid-state conversion, (iii) have structural compatibility and lattice matching, which reduces interfacial defects caused by lattice mismatch and minimizes interfacial stress, thereby enhancing the long-term durability and ion selectivity.

In general, an ideal SRL serves as a protective barrier to achieve a balance between the deceleration of the direct contact of H^+^ ions with the MnO_2_ and the dissolution process of Mn species. Simultaneously, it regulates the dynamic equilibrium of multiple ionic concentrations (e.g. H^+^, Mn^2+^) within the interfacial microenvironment, ensuring localized ion homeostasis that kinetically restricts the growth duration. Additionally, the abundant surface adsorption sites of the SRL facilitate the nucleation process of reaction products through preferential binding interactions, which suppresses continuous ion aggregation around embryonic nuclei in the electrolyte. This dual control reduces the average precipitate size, demonstrating precise size modulation between the solid-phase ZHS and ZMO. This strategy surpasses traditional surface microenvironment regulation methods in the MnO_2_ cathode (Fig. 1c and Table S1), offering greater flexibility and broader applicability in interphase-layer design.

Among the various SRLs, we rationally selected bismuth oxide (Bi_2_O_3_) because of the robust interactions with Zn (Fig. 1d) and strong affinity on MnO_2_ [26]. It contributed to the formation of abundant ZHS and ZMO nucleation sites on the surface. Furthermore, Bi_2_O_3_ demonstrates notable buffering effects on the Mn^2+^ and H^+^ during the initial cycles, stabilizing the ionic concentrations and enabling controlled product growth. This control efficiently promotes small-sized solid–solid conversion between the ZHS and ZMO phase in extended cycles, steering the reaction pathway of the solid phase towards high reversibility. The iron-plate batteries with SRLs achieve a specific capacity of 401.8 mAh g^−1^ at 0.2 C, with cathode mass loading of >9 mg cm^−2^. Even with a material mass loading amplified to >16 mg cm^−2^, the battery maintains stable operation for >110 days (equivalent to 350 cycles) at 0.2 C. This advancement can be extended to other cathode materials, such as Prussian blue analogs, vanadium-based oxides and polyanion-type compounds, which will trigger a wave of investigation on the precise regulation of solid-phase conversion reactions in aqueous batteries and beyond.

## RESULTS AND DISCUSSION

### Design principles of SRLs

**Figure 2.**
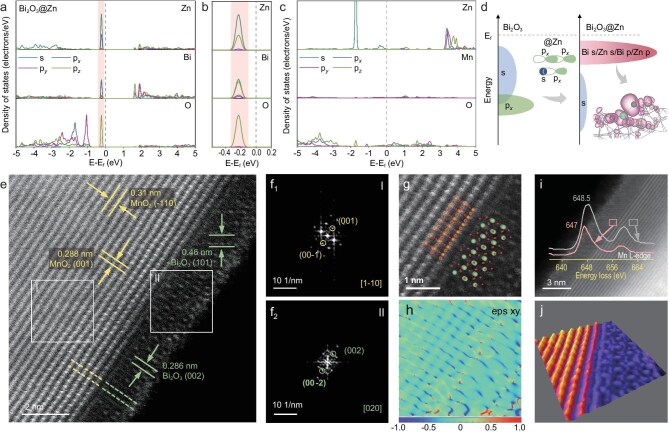
Design and structure of the SRLs. (a) DOS curves of Bi_2_O_3_@Zn showing the hybridization between the Zn and Bi/O atoms in Bi_2_O_3_. (b) Enlarged view of (a) marked in pink. (c) DOS curves of MnO_2_@Zn. (d) Schematic diagram of electron–orbit coupling in Bi_2_O_3_@Zn. (e) Spherical AC-high-angle annular dark-field scanning transmission electron microscopy (HAADF-STEM) image of MnO_2_@BiO nanorod and (f) corresponding fast Fourier transform of regions I and II in Fig. 2e. (g) HAADF-STEM image of the epitaxial interface between MnO_2_ along ⟨1–10⟩ and Bi_2_O_3_ along ⟨020⟩ of MnO_2_@BiO. (h) ε*_xy_* strain map obtained by conducting geometric phase analysis of (g). (i) HAADF-STEM image of the epitaxial interface and corresponding electron energy loss spectroscopy Mn-L2,3 edges of the region marked in left and right of MnO_2_@BiO. (j) 3D atom-overlapping Gaussian-function fitting map of (g).

The dissolution–redeposition dynamics of MnO_2_ cathodes in aqueous zinc-ion batteries necessitate phase-specific regulation through buffer-layer engineering. This work proposes an SRL architecture that synergistically regulates both the thermodynamic stability of MnO_2_ and the kinetic landscape for controlled product deposition. Through the systematic screening of d-/p-block metal oxides by using density functional theory (DFT), we evaluated SRL candidates by considering adsorption energy of Zn. Intriguingly, Bi_2_O_3_ demonstrated exceptional Zn-adsorption characteristics, with a calculated adsorption energy (*E*_ads_) of −4.48 eV on the (100) facet (Fig. 1d and Fig. S1a), suggesting the robust interactions with Zn during the initial discharge. The nucleation of products on the substrate surface must overcome an energy barrier [27]. The strong chemical affinity between Bi and Zn can markedly lower the activation energy for ZHS nucleation on the Bi_2_O_3_ surface, triggering heterogeneous nucleation and increasing the ZHS nucleation density [28]. Therefore, this strong interfacial interaction promotes the heterogeneous nucleation of ZHS on the cathode surface [29]. Chronoamperometry curves of Bi_2_O_3_-coated MnO_2_ (MnO_2_@BiO) (Fig. S2) show that Bi_2_O_3_ accelerates the initial nucleation of ZHS due to increased nucleation sites and an accelerated instantaneous deposition rate, corroborating that the Bi_2_O_3_ reduces the nucleation barrier and shortens the nucleation period. Electronic structure analysis via density of states (DOS) further reveals that the enhanced Zn affinity originates from a synergistic σ-bonding interaction between Zn s/p_*x*_ orbitals and Bi s/p*_x_* orbitals (Fig. 2a, b and d, and Table S2). In contrast, pristine MnO_2_ exhibits negligible orbital coupling with Zn (Fig. 2c and Figs S1b and S3), resulting in inferior interactions stabilization. The number of crystal facets with <5% mismatch between each oxide-phase crystal facet and the MnO_2_ (001) was counted by using standard phase cards (Fig. S4) and the highest number of possible matches was found in Bi_2_O_3_, implying the most probable lattice compatibility. Collectively, these insights establish Bi_2_O_3_ as the optimal SRL candidate for enhancing interfacial kinetics, which directly correlates with the superior electrochemical performance metrics of Bi_2_O_3_-modified cathodes discussed in the subsequent section. These findings underpin a dual-function design strategy of surface-engineered SRLs: (ⅰ) adsorption-facilitated nucleation leveraging strong Zn-bonding sites that directs optimal phase evolution and reaction pathway; (ⅱ) lattice-matched epitaxial coating-induced dynamic dissolution control that achieves optimal Mn^2+^ flux and maintains the microenvironment stability that is favorable for small-sized solid deposition. Based on these design principles, we constructed a Bi_2_O_3_-based SRL architecture on an MnO_2_ surface and its composite structure was analysed in detail.

MnO_2_ cathodes incorporating Bi_2_O_3_ were synthesized via aqueous-phase *in situ* encapsulation followed by controlled thermal annealing. High-resolution X-ray diffraction (XRD) analysis in the range of 25°–35° 2*θ* (Fig. S5) confirms the coexistence of bismuth oxide (*β*-Bi_2_O_3_, PDF#27–0050) through distinctive (201) and (220) diffraction patterns. The modified MnO_2_ retains the pristine crystallographic phase while preserving its characteristic nanorod morphology (Figs S6 and S7a). High-angle annular dark-field scanning transmission electron microscopy coupled with elemental mapping demonstrates homogeneous bismuth (Bi) distribution across the MnO_2_ nanorod surface, as validated by using cross-sectional line scanning profiles (Fig. S7a). X-ray photoelectron spectroscopy (XPS) of Bi 4f orbitals further corroborates successful Bi incorporation (Fig. S8). A crystallographic registry between MnO_2_ and *β*-Bi_2_O_3_ is established through comparative lattice imaging, in which contrast variations arise from differential electron transmittance between the nanorod core (brighter MnO_2_ regions) and surface-modified zones (darker *β*-Bi_2_O_3_ domains) (Fig. 2e). This contrast originates from the inherent thickness gradient between the periphery and core regions of the nanorod. Lattice parameter analysis and fast Fourier transform deconvolution reveal epitaxial alignment between the MnO_2_ {001} and Bi_2_O_3_ {002} planes (Fig. 2f). Distinct bright/dark atomic-scale variations within the coating layer clearly indicate the intermixing of Bi atoms and Mn atoms (Fig. S9). Energy-dispersive X-ray spectroscopy (EDS) line scanning of MnO_2_@BiO (Fig. S7b) also indicates the diffusion of Mn into the Bi_2_O_3_. As the diffusion coefficient of ions in the solid is several orders of magnitude slower than that in the liquid, we utilize the transfer of Mn^2+^ in the solid to slow down the diffusion of Mn^2+^ into the electrolyte due to the concentration gradient, suppress the excessive dissolution of Mn during the initial discharge process and maintain the necessary dissolution–deposition electrochemistry simultaneously. Such crystallographic continuity facilitates regulated Mn^2+^-ion migration, establishing ion-conducting channels. Therefore, we have established a regulatory pathway rather than an obstructive one, aiming to prevent the disordered diffusion of large amounts of Mn^2+^. Furthermore, this lattice-matched configuration enhances electronic percolation networks, effectively reducing interfacial impedance [30], and promotes the growth and deposition of interfacial products [31]. Systematic Bi loading optimization identifies 5 wt% as the critical threshold beyond which elemental segregation occurs, evidenced by secondary phase formation (Fig. S10). Bi_2_O_3_ buffer layers (thickness range of 1.5–2.5 nm) were evaluated by using coin-cell testing and a thickness of 2 nm (3 wt% Bi) was identified as being optimal for ion-transport efficiency (Fig. S11). High-resolution Bi 4f XPS (Fig. S12) tests were conducted on MnO_2_@BiO materials with thicknesses of 1.5–2.5 nm Bi_2_O_3_, which also verified the atomic mixture of Mn and Bi in the Bi_2_O_3_ layer. When Mn^2+^ ions transfer in the Bi_2_O_3_, the doped Mn also exerts a certain repulsive force on the Mn^2+^, playing a role in deceleration. Comparative studies with alternative metal-oxide modifiers (Figs S13 and S14) reveal inferior Zn interaction kinetics, correlating with their limited cycling stability, as detailed in the subsequent section. Atomic-scale structural analysis (Fig. 2g) combined with *xy*-plane geometric phase analysis demonstrates near-zero interfacial strain, confirming stress-free heterointerface formation (Fig. 2h). Electron energy loss spectroscopy across the phase boundary (Fig. 2i, gray/red zones) reveals a modified Mn L-edge fine structure, indicating electron-transfer-induced Mn-valence reduction. Mn may also diffuse into the Bi_2_O_3_ lattices during annealing, forming Mn–O–Bi coordination complexes (Fig. 2j, between the large-sized Bi atoms on the right side), and thus the dissolution resistance for Mn^2+^ remains within the optimal thresholds. This atomic intermixture and moderate layer thicknesses provide the basis for maintaining essential dissolution reaction and redox chemistry. The resultant MnO_2_@BiO architecture establishes a self-regulated interfacial microenvironment that governs the product nucleation kinetics and morphology evolution.

### Modulatory effects of the size-revitalizing layers in the initial cycles

MnO_2_@BiO and pristine MnO_2_ cathodes were integrated into coin-type configurations to assess the modulatory effects of the SRL on interfacial ionic environments and reaction product evolution. Coin cells were fabricated by using a Zn-foil anode and 2 M ZnSO_4_/0.2 M MnSO_4_ electrolyte. Parallel galvanostatic charge–discharge (GCD) profiles and mass variation trends monitored via an electrochemical quartz crystal microbalance (EQCM) (Fig. S15) demonstrate preserved MnO_2_ cathode reaction mechanisms post Bi_2_O_3_ modification. Distinctive disparities in the first-cycle discharge curves and cyclic voltammetry (CV) profiles and mass fluctuations between initial versus subsequent cycles (Figs S15 and S16) reveal unique electrochemical signatures during battery activation. To accurately elucidate the cathode behavior in the mildly acidic electrolyte, we systematically probed the phase evolution through: (ⅰ) *in situ* XRD tracking of crystalline transformations (Fig. S17), (ⅱ) *ex situ* XPS of elemental states across the discharge/charge phases (Fig. S18) and (ⅲ) stage-correlated EQCM mass transport analysis (Fig. S15). Synthesis of these datasets establishes the first discharge sequence as: MnO_2_ dissolution initiating from proton-insertion pathways and the sequential formation of MnOOH and ZHS. Subsequent charging processes involve the ZHS decomposition and codeposition of Zn^2+^/Mn^2+^, forming ZMO. The post-first-cycle behavior transforms to multistep processes, involving sustained MnO_2_ dissolution, ZHS generation and ZMO decomposition during discharge, followed by ZHS dissolution and Zn–Mn codeposition during charging. EDS and high-resolution transmission electron microscopy (HRTEM) characterization of MnO_2_@BiO at various cutoff voltages (Figs S19 and S20) confirm the ZHS dissolution/ZMO reformation dynamics. Upon complete MnO_2_ consumption (Fig. S21), the system evolves into a reversible ZHS↔ZMO interconversion. The reaction equations on the cathode are shown in Equations (1–6). Crucially, interfacial ion-transport kinetics governs the dissolution–deposition equilibrium, profoundly influencing the nucleation thermodynamics, phase growth trajectories and ultimate reaction pathway.

Discharge:


(1) \begin{eqnarray*}
&& {\mathrm{Mn}}{{\mathrm{O}}}_2 + {\mathrm{ 2}}{{\mathrm{H}}}^ + + {\mathrm{ 2}}{{\mathrm{e}}}^ - \\
&&\quad \to {\mathrm{M}}{{\mathrm{n}}}^{2 + } + {\mathrm{ 2O}}{{\mathrm{H}}}^ - \left( {{\mathrm{until\ Mn}} {{\mathrm{O}}}_{\mathrm{2}}\ {\mathrm{vanished}}} \right),\quad
\end{eqnarray*}

(2) \begin{eqnarray*}
&&{\mathrm{Mn}}{{\mathrm{O}}}_2 + \ {{\mathrm{H}}}^ + + \ {{\mathrm{e}}}^ -\\
&&\to {\mathrm{MnOOH }}\left( {{\mathrm{until\ Mn}}{{\mathrm{O}}}_{\mathrm{2}}\ {\mathrm{vanished}}} \right),
\end{eqnarray*}

(3) \begin{eqnarray*}
&&4{\mathrm{Z}}{{\mathrm{n}}}^{2 + } + {\mathrm{ S}}{{\mathrm{O}}}_4{^2}^ - + \ 6{\mathrm{O}}{{\mathrm{H}}}^ - + \ {{\mathrm{H}}}_{\mathrm{2}}{\mathrm{O}}\\
&&\to {\mathrm{Z}}{{\mathrm{n}}}_4{\mathrm{S}}{{\mathrm{O}}}_4{\left( {{\mathrm{OH}}} \right)}_6\cdot{{x}}{{\mathrm{H}}}_{\mathrm{2}}{\mathrm{O}},
\end{eqnarray*}

(4) \begin{eqnarray*}
&&{\mathrm{ZnM}}{{\mathrm{n}}}_{{x}}{{\mathrm{O}}}_{{y}} + {{y}}{{\mathrm{H}}}^ + + {{2x}}{{\mathrm{e}}}^ - \\
&&\quad \to {\mathrm{Z}}{{\mathrm{n}}}^{2 + } + {x\mathrm{ M}}{{\mathrm{n}}}^{2 + } + {y\mathrm{ O}}{{\mathrm{H}}}^ - \left( {{\mathrm{from\ 2nd\ cycle}}} \right).\\
\end{eqnarray*}

Charge:


(5) \begin{eqnarray*}
&&{\mathrm{Z}}{{\mathrm{n}}}_4{\mathrm{S}}{{\mathrm{O}}}_4{\left( {{\mathrm{OH}}} \right)}_6\cdot{{x}}{{\mathrm{H}}}_2{\mathrm{O}}\\
&&\to 4{\mathrm{Z}}{{\mathrm{n}}}^{2 + } + {\mathrm{ S}}{{\mathrm{O}}}_4{^2}^ - + \ 6{\mathrm{O}}{{\mathrm{H}}}^ - + \ {{\mathrm{H}}}_2{\mathrm{O}},
\end{eqnarray*}

(6) \begin{eqnarray*}
&&{\mathrm{Z}}{{\mathrm{n}}}^{2 + } + {x\mathrm{ M}}{{\mathrm{n}}}^{2 + } + {y\mathrm{ O}}{{\mathrm{H}}}^ - \\
&&\to {\mathrm{ZnM}}{{\mathrm{n}}}_x{{\mathrm{O}}}_y + {{y}}{{\mathrm{H}}}^ + + 2{{x}}{{\mathrm{e}}}^ - .
\end{eqnarray*}

**Figure 3.**
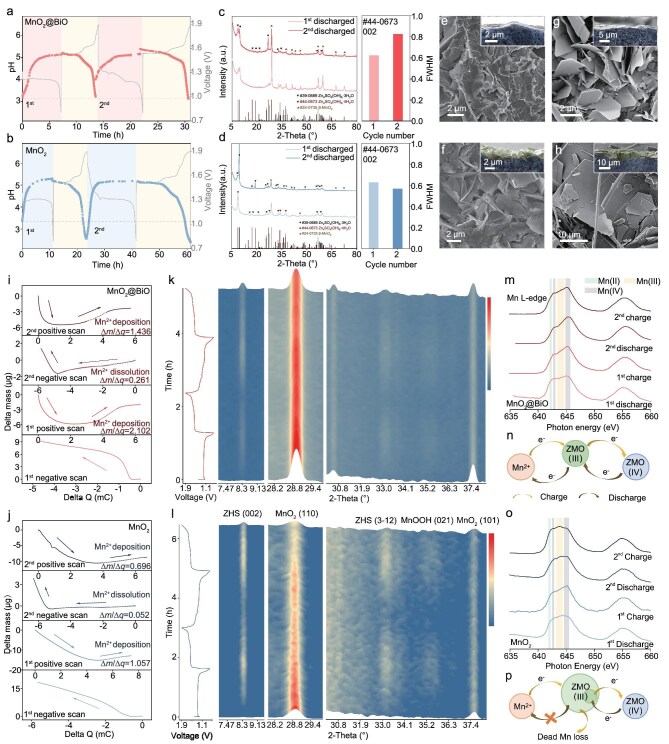
Modulatory effects of the SRL in initial cycles. (a, b) Galvanostatic charge and discharge profiles of Zn–Mn batteries and the corresponding graph of *in situ* pH monitoring from the first discharge to the second charge (0.1 C, 2 M ZnSO_4_/0.2 M MnSO_4_) of MnO_2_@BiO and MnO_2_. (c, d) *Ex situ* XRD patterns of MnO_2_@BiO and MnO_2_ after the first and second full discharges. (e, g) SEM images of the MnO_2_@BiO cathode after the first and second full discharges. (f, h) SEM images of the MnO_2_ cathode after the first and second full discharges. The insets are cross sections of the cathodes. (i, j) Mass change-charge profiles of MnO_2_@BiO and MnO_2_ cathodes in the first and second CVs. (k, l) *In situ* XRD patterns of the MnO_2_@BiO and MnO_2_ cathodes in the first and second cycles. (m, o) Soft X-ray absorption spectroscopy of the MnO_2_@BiO and MnO_2_ cathodes at the first and second fully discharged and charged states. (n, p) Schematic of the charging process of the MnO_2_@BiO and MnO_2_ electrodes.

The introduction of Bi_2_O_3_ as a prototypical SRL onto MnO_2_ effectively suppresses the direct contact between H^+^ and MnO_2_ and the drastic leaching of Mn^2+^ ions into the electrolyte during the initial discharge. Figure S21 shows that, for MnO_2_@BiO, the diffraction intensity of the MnO_2_ phase continuously weakened within 50 cycles; the MnO_2_ phase was still identifiable after the 50th cycle but had completely disappeared by the 100th cycle. In contrast, the MnO_2_ phase in the bare MnO_2_ electrode vanished after only 20 cycles, confirming its severe structural instability during the initial cycling. The buffer-mediated modulation establishes a stabilized cathode/electrolyte interface with mitigated chemical gradients. Operando pH monitoring via a dual-electrode system (Fig. S22) reveals that MnO_2_@BiO exhibits controllable interfacial alkalization during discharging and maintains reversible pH changes after cycles (Fig. 3a and b). Bi_2_O_3_ acts as a physical barrier, undertaking partial H^+^ intercalation, avoiding the disorderly intercalation of protons into the MnO_2_ lattice within a short time. First, Bi–O sites on the Bi_2_O_3_ surface can capture some protons. Under acidic conditions, the surface Bi–O bonds act as proton acceptors, with O^2−^ becoming Lewis basic sites that bind H^+^ to form OH. Subsequently, H^+^ enters MnO_2_ through the Bi_2_O_3_ lattice, thereby achieving the effect of slowing down and diffusing within the solid phase. The buffered proton consumption kinetics creates a controlled enrichment of hydroxyl ions. The increased ZHS nucleation density due to the strong interactions between Zn and Bi, in combination with the low OH^−^ concentration, will lead to the small-sized growth and restrict excessive precipitation of ZHS [32–34]. Comparative structural analysis reveals evidence of modified crystallization behavior. The characteristic XRD patterns (Fig. 3c and d) show attenuated ZHS diffraction intensities (PDF#39–0689, 44–0673) in the MnO_2_@BiO cathodes compared with the pristine MnO_2_. Comparison of the full width at the half maxima of (002) also reveals a decrease in the ZHS growth size. ZHS typically exhibits hexagonal plate morphology in scanning electron microscopy (SEM) images due to the triclinic structural and layered properties (Fig. 3e–h). The planar and cross-sectional images of discharged cathodes also confirm significant difference in ZHS growth. The radial dimensions of ZHS formed on the MnO_2_@BiO are an order of magnitude smaller in size than those on pristine MnO_2_. Additionally, the intensity of the *β*-MnO_2_ characteristic patterns decreases less in fully discharged MnO_2_@BiO, corresponding to the slower release of Mn ions, and the Mn concentration in the electrolyte detected by using inductively coupled plasma (ICP) analysis shows a reduction in Mn dissolution in the MnO_2_@BiO (Fig. S23). The SRL acts as a physical barrier regulating ion transport and establishes a self-limiting microenvironment in the initial discharge process. The SRL restricts ZHS overgrowth through kinetic confinement, maintains MnO_2_ structural integrity via dissolution inhibition and facilitates subsequent ZHS decomposition through optimized crystallite dimensions.

The dissolution and codeposition pathways of Mn species related to electron transfer on the cathodes were systematically investigated by using EQCM (Fig. 3i and j, and Fig. S24). The mass-to-charge ratio (Δ*m*/Δ*Q*) obtained during the posterior segment of the positive scan and the anterior segment of the negative scan reveal Zn–Mn codeposition characteristics and Mn dissolution, respectively [20]. A higher slope signifies more complete electron participation. The first negative-scan profiles show that the overall electrode mass increases and the stages corresponding to MnO_2_ dissolution and ZHS formation cannot be resolved because the two processes occur simultaneously. Consequently, the mass increase recorded by using EQCM during the first negative scan cannot be ascribed to the dissolution of MnO_2_. Notably, the MnO_2_@BiO cathode exhibits twice the mass accumulation per unit charge compared with pristine MnO_2_ (Δ*m*/Δ*Q* = 2.102 vs. 1.057 μg/mC, Δ*m*/Δ*Q* = 1.436 vs. 0.696 μg/mC) during the first and second Mn depositions, coupled with a reduced interfacial ion concentration, suggesting enhanced deposition completeness. Meanwhile, the comparison of the mass-to-charge ratio representing the Mn dissolution reveals that the solid-to-ion conversion in MnO_2_@BiO is more complete (Δ*m*/Δ*Q* = 0.261 vs 0.052 μg/mC). Consequently, the MnO_2_@BiO electrode is demonstrated to promote the Mn deposition/dissolution reactions more effectively. Such mechanistic differences enable the SRL to prevent incomplete electrochemical reactions while establishing efficient electron-transfer pathways. The adequacy of the electrochemical reactions also affects the state of the product [23]. *In situ* XRD analysis (Fig. 3k and l) was used to track the phase-evolution dynamics, with characteristic diffraction patterns corresponding to the ZHS, MnO_2_ and MnOOH phases. Comparative intensity variations between the ZHS (002) and MnO_2_ (110) diffraction peaks reveal the SRL-mediated regulation of crystalline product growth and Mn-dissolution kinetics. Notably, incomplete MnOOH decomposition in pristine MnO_2_ during the second cycle (vs. first cycle) suggests that Mn^2+^ supersaturation in the electrolyte impedes dissolution equilibrium. Morphological analysis demonstrates that the MnO_2_@BiO cathodes form uniform submicron flakes versus micron-scale irregular deposits on the pristine MnO_2_ (Fig. S25). Both systems exhibit amorphous characteristics post charging (Figs S21c and d and S26), consistent with their low crystallinity indices [35]. Reaction kinetics analysis reveals that pristine MnO_2_ undergoes rapid structural evolution, with interfacial microenvironment differences inducing divergent morphological and oxidation state outcomes [36,37]. X-ray absorption spectroscopy (XAS) (Fig. 3m and o) and XPS (Fig. S27) analysis of Mn speciation show reduced Mn(Ⅲ) intensities in MnO_2_@BiO at both discharged and charge states compared with pristine MnO_2_. This aligns with the established role of Mn(Ⅲ) as electrochemically inert intermediates [13,14,38], whose accumulation is suppressed through SRL-enabled dynamic Mn^2+^-concentration control. The modulation drives complete conversion to Mn(Ⅳ) products while minimizing parasitic intermediate formation. In the sulfate-based electrolyte (pH ≈ 4), Mn^2+^ ions are oxidized to Mn(IV) through a solid-state reaction involving an Mn(III) intermediate [39]. During the initial charging cycles, Zn^2+^ and Mn^2+^ ions deposit as ZMO(III) on the electrode surface. This process is diffusion-controlled, analogous to ZHS growth, but with the additional step of electron transfer. The strong interaction between Bi and Zn, which increases the nucleation site density, also facilitates ZMO nucleation. Concurrently, due to the physical-barrier effect of Bi_2_O_3_, fewer Mn^2+^ ions dissolve, resulting in a lower concentration of Mn^2+^ in the electrolyte. Consequently, during the initial ZMO deposition, the ZMO(III) particles on the MnO_2_@BiO electrode are smaller than those on the bare MnO_2_ electrode. Transport resistance can be readily reduced by using nanosized active materials and thinner composite electrodes, enabling faster electron transfer [40]. The smaller ZMO(III) particles exhibit shorter electron-transport pathways in subsequent electrochemical oxidation, inducing a more complete electrochemical reaction (Fig. 3n and Fig. S28a). Within the bare MnO_2_ electrode, Mn(III) sites located deep within large particles struggle to contact the electrode/current collector interface and suffer from sluggish electron/ion transport. This prevents them from acquiring sufficient charge for complete oxidation from Mn(III) to Mn(IV). Consequently, Mn(III) accumulates internally as incompletely reacted residues (Fig. 3p and Fig. S28b).

### Size-dependent solid–solid conversion in extended cycling

**Figure 4.**
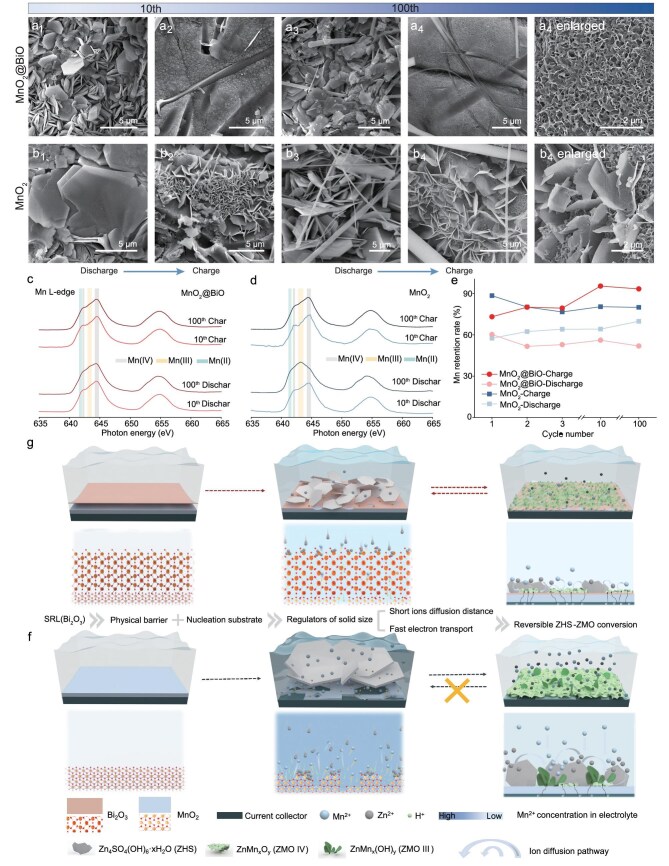
Modulatory effects of the SRL induced in extended cycling. (a1–a4) SEM images of the MnO_2_@BiO cathode at 10th and 100th discharged and charged states. (b1–b4) SEM images of MnO_2_ cathode at 10th and 100th discharged and charged states. (c, d) *Ex situ* XAS of the MnO_2_@BiO and MnO_2_ cathodes at 10th and 100th discharged and charged states. (e) Mn-retention rate evolution in increasing cycles of MnO_2_@BiO and MnO_2_ cathode testing using ICP. (f, g) Schematic diagram of SRL regulation effects.

Dynamic battery processes exhibit characteristics of chaotic systems and the degradation of the electrode is a function of time [41] in which minor deviations in the initial conditions can lead to significant long-term behavioral variations and can be expressed as ‘sensitive dependence on initial conditions’ (SDIC). By modulating the interfacial microenvironment during the initial cycling process, we aim to achieve long-term regulation of the product size, thereby enhancing the cycling stability of the Zn–Mn battery. This approach aligns precisely with the concept expressed by SDIC. As previously discussed, the influence of the initial cathode/electrolyte interface on the intricate interplay of chemical and electrochemical reactions has been preliminarily observed. The SRL mediates the diffusion of controlled ions during early cycling, establishing a stable interfacial environment that supports subsequent complex reactions in the Zn–Mn battery. As illustrated in Fig. S29, the Bi_2_O_3_ layer shows no significant chemical dissolution or electrochemical reduction during the initial cycles (evidenced by its intact interface), effectively accommodating volumetric changes. After long-term cycles, the original MnO_2_ appears to be completely dissolved and/or restructured. Meanwhile, Bi_2_O_3_ persists in the cathode but exhibits substantial morphological and distribution changes (Fig. S30). Beyond the restructuring stage, the primary roles of physical barrier and hetero-growth substrate ceased. Crucially, the stabilized interfacial microenvironments and small-sized solid phases formed under the guidance of Bi_2_O_3_ during early cycles lay the foundation for long-term stability. Thus, in later cycling stages, the state of Bi_2_O_3_ became noncritical for maintaining stability, as the favorable microenvironment established earlier ensured sustained system performance. A favorable microenvironment persisted throughout the extended cycle, maintaining a consistent ZHS-to-ZMO solid–solid conversion mechanism (Figs S31–S33). Post-cycling morphology comparisons underscore the sustained impact of the SRL (Fig. 4a and b). After the 10th discharge, the MnO_2_@BiO cathodes display uniformly distributed ∼1-μm ZHS flakes (Fig. 4a_1_), contrasting with the 10–20-μm flakes on the pristine MnO_2_ (Fig. 4b_1_), reflecting a 10-fold size difference in discharged products. MnO_2_@BiO further demonstrates a homogeneous deposition (Fig. 4a_2_), whereas pristine MnO_2_ forms aggregated large-sized flakes (Fig. 4b_2_), identified as Zn/Mn codeposits. With the progression of cycling, after 100 cycles, the distinction between the two cathodes remains pronounced (Fig. 4a_3_ and b_3_), with analogous disparities in the charged products (Fig. 4a_4_ and b_4_). High-magnification SEM of the 100th fully charged MnO_2_ reveals small products alongside micron-sized flakes (Fig. 4a_4_ and b_4_ enlarged), which exceed the dimensions observed after the 10th cycle. These differences are accentuated under broader imaging scales (Fig. S34). These larger flakes originate from ZMO(Ⅲ) accumulation, exacerbating cathode degradation. Conversely, the SRL promotes continuous solid–solid conversion between small-sized ZHS and ZMO, indicating enhanced reaction reversibility.

Our findings demonstrate that the initial cathode/electrolyte interface critically determines the long-term reaction pathways through the SRL-mediated regulation of Mn^2+^ flux and product size-dependent reaction kinetics. We employed XAS and XPS (Fig. 4c and d, and Fig. S35) to track the Mn states in the cycled cathodes. In the MnO_2_@BiO cathode, the persistent dominance of the Mn(IV) state during cycling indicates stable solid–solid conversion, showing uniform coverage of small-sized ZMO deposits in the charged state. The charging process involves Mn^2+^ deposition as ZMO(III) particles followed by electrochemical oxidation to ZMO(IV). Under the long-term effects of the incomplete conversion of large-sized products, Mn(III) states were presented in the bare MnO_2_ cathode at the 100th complete discharge, indicating the accumulation of electrochemically inert substances. In its corresponding fully charged state, the Mn(IV) state suggests the loss of inert Mn(III), as illustrated in Fig. 3p. Large ZMO(III) deposits on the bare cathode show uneven electron distribution: the inner layer in direct contact with the conductive carbon readily receives electrons and undergoes reductive dissolution, while dissolution of the outer layer is delayed due to insufficient electron supply. Once the inner layer dissolves, the remaining ZMO(III) detaches and precipitates into the electrolyte. This contrast highlights the critical role of SRL engineering in regulating interfacial processes. The SRL-stabilized system maintains nanoscale product dimensions, minimizing the mechanical strain and phase-boundary resistance that trigger the degradation in conventional cathodes. ICP analyses (Fig. 4e) further corroborate this stabilization, with MnO_2_@BiO exhibiting sustained Mn-retention hysteresis in contrast to the progressive Mn depletion observed in the unmodified cathodes. The retention metrics, calculated as the mass ratio of the post-cycled to pristine manganese content, reveal enhanced reversibility in SRL-modified systems. The difference between the charged and discharged states quantifies the deposition/dissolution efficiency, showing 87% reversibility for MnO_2_@BiO versus 52% for the control group after 100 cycles.

As shown in Fig. 4f and g, Bi_2_O_3_ first acts as a physical barrier reducing the Mn²⁺ and H⁺ concentrations on the interface. The Bi–O sites on Bi_2_O_3_ capture protons, after which H⁺ migrates into the solid Bi_2_O_3_ lattice towards the MnO_2_ surface via the Grotthuss mechanism [42,43], hopping through a hydrogen-bonded oxygen network (Fig. S36a and b). Mn^2+^ migration primarily relies on intrinsic oxygen vacancies in Bi_2_O_3_ [44] and lattice defects from Mn doping, where vacancies serve as critical ‘hopping sites’ for vacancy-assisted ion transport under electric fields/concentration gradients [45] (Fig. S36c and d). This solid-state transport significantly suppresses the disordered diffusion of Mn^2+^ into the electrolyte and excessive H^+^ insertion. Secondly, Bi_2_O_3_ serves as a nucleation substrate for products, increasing the nucleation density and synergizing with low ion concentrations to induce the formation of small-sized ZHS and ZMO deposits. Leveraging optimized ion/electron-transport paths enabled by this small-size effect, a reversible Mn(II)–Mn(IV) pathway is achieved. A detailed comparison of the reaction process can be seen in Fig. S37. The size-dependent solid–solid conversion promoted by the SRL minimizes volume changes and material loss. These synergistic effects collectively enable exceptional cycling stability for Zn–Mn batteries under harsh conditions, including high mass loading and prolonged cycling.

### Extended cycling stability of batteries with high-mass-loading cathodes/scalable battery for practical energy storage

**Figure 5.**
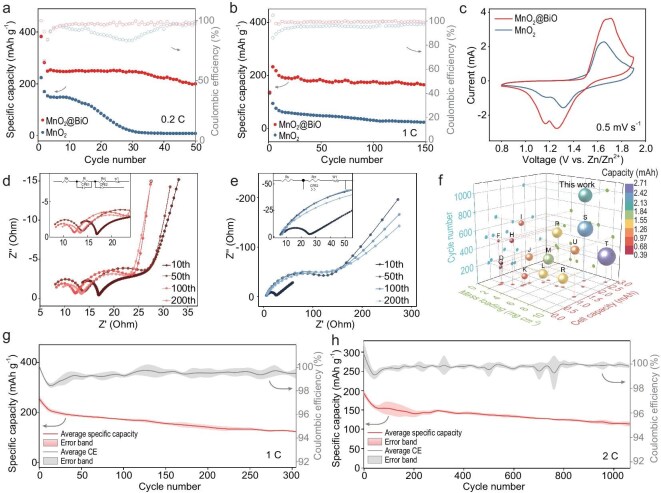
Cycling performance of high-mass-loading MnO_2_@BiO cathode in coin batteries. (a, b) Cycling performance of the MnO_2_ and MnO_2_@BiO cathodes in 2 M ZnSO_4_/0.2 M MnSO_4_ electrolyte at 0.2 and 1 C. The active material mass loading is ∼9 mg cm^−2^. (c) CV profiles of MnO_2_ and MnO_2_@BiO cathodes in the third cycle, with a scan rate of 0.5 mV s^−1^. (d, e) Nyquist plots of the MnO_2_ and MnO_2_@BiO cathodes in 2 M ZnSO_4_/0.2 M MnSO_4_ electrolyte over the frequency range of 100 mHz–1 MHz at selected cycles. Inset is the corresponding equivalent circuit diagram. (f) Comparison of cell-cycle number, active material mass loading with recently reported coin-type Zn–Mn batteries. More details are listed in Table S5. (g, h) Cycling performance of the MnO_2_@BiO cathode in 2 M ZnSO_4_/0.2 M MnSO_4_ electrolyte at 1 and 2 C. Standard deviations in these plots are calculated based on three individual cells (1 C = 250 mA g^−1^).

Electrochemical evaluation across coin-type and large plate cells demonstrates the superior interfacial stabilization achieved through SRL engineering. At 0.2 C with active material loading of >9 mg cm^−2^, the MnO_2_@BiO cathode retains 200 mAh g^−1^ after 50 cycles, while pristine MnO_2_ suffers 61% capacity decay within 20 cycles (Fig. 5a). Furthermore, the MnO_2_ cathode exhibits a significant voltage plateau decline within 50 cycles (Fig. S38), reflecting progressive deterioration. In contrast, the MnO_2_@BiO cathode shows stable discharge plateaus and excellent capacity retention over repeated cycles. This performance divergence intensifies under accelerated testing conditions, where the modified cathode maintains ∼83% capacity retention over 150 cycles at 1 C, coupled with coulombic efficiencies of >99.5% (Fig. 5b). CV profiles reveal fundamental mechanistic differences: the MnO_2_@BiO cathode exhibits redox peak symmetry with a 77% higher peak response current compared with that of the control group (Fig. 5c), confirming enhanced reaction kinetics and electrochemical reversibility due to SRL optimization. Nyquist plots elucidate the degradation pathways, showing a charge-transfer resistance (*R*_ct_) escalation from 16.7 to 147 Ω over 200 cycles for pristine MnO_2_ (Fig. 5e and Table S3), possibly due to the formation of ZMO (Mn, III), which impedes the charge-transfer processes. In contrast, the MnO_2_@BiO cathode (Fig. 5d) maintains stable *R*_ct_ values (12.5 ± 3.9 Ω) with additional semicircular features corresponding to optimized interfacial impedance (*R*_i_), demonstrating the dual functionality of the SRL in charge-transfer mediation and interface stabilization. Comparative studies with Al_2_O_3_- and TiO_2_-modified cathodes reveal critical design constraints—while MnO_2_@TiO initially exhibits comparable redox activity, its rapid failure within 20 cycles at 1 C (Fig. S39) underscores the unique chemical compatibility of Bi_2_O_3_ in maintaining long-term interfacial integrity. A systematic comparison of the three materials is shown in Figs S40–S44 and Note S1.

Statistical analysis of cycling reproducibility across three independent cells demonstrates exceptional consistency, with capacity deviations constrained to ±6.5 mAh g^−1^ over 200 cycles at 1 C (Fig. 5g). The system also achieves breakthrough longevity metrics, retaining 85% of the initial capacity after 1000 cycles at 2 C with coulombic efficiency averaging 99.8 ± 0.2% (Fig. 5h). Specific capacities and coulombic efficiency data for Zn–MnO_2_@BiO cells are detailed in Fig. S45 and Table S4. Furthermore, this performance holds the potential to translate directly into practical applications, delivering 1.96 mAh at 9 mg cm^−2^ of mass loading with 1000 cycles—a 140% improvement in lifespan over those of conventional counterparts (Fig. 5f and Table S5). Such enhancement is attributed to the SRL-mediated preservation of the ZHS–ZMO conversion pathways, reducing capacity loss and bolstering long-term stability through the suppression of irreversible phase transformations.

**Figure 6.**
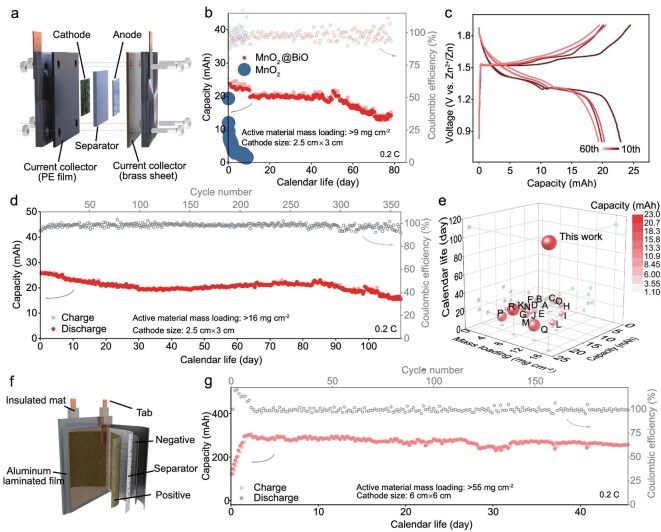
Cycling performance of high-mass-loading MnO_2_@BiO cathode in large batteries. (a) Internal view of the plate battery. (b) Cycling performance of the MnO_2_ and MnO_2_@BiO cathodes in 2 M ZnSO_4_/0.2 M MnSO_4_ electrolyte at 0.2 C. The active material mass loading is >9 mg cm^−2^ and the N/P ratio is 2.560. (c) Corresponding charging/discharging plateaus. (d) Cycling performance of the MnO_2_@BiO cathode in 2 M ZnSO_4_/0.2 M MnSO_4_ electrolyte at 0.2 C. The active material mass loading is ∼16 mg cm^−2^. (e) Comparison of cell calendar life, active material mass loading and cell capacity with recently reported zinc-ion batteries. More details are listed in Table S6. (f) Internal view of the pouch-type cell. (g) Cycling performance of the ε-MnO_2_@BiO cathode in a pouch-type cell in 2 M ZnSO_4_/0.2 M MnSO_4_ electrolyte at 0.2 C. The active material mass loading is >55 mg cm^−2^.

The development of large-format battery systems (Fig. 6a) necessitates an electrode that is capable of maintaining electrochemical fidelity under industrially relevant conditions. Our systematic evaluation of MnO_2_@BiO cathodes in scaled configurations reveals exceptional performance metrics that are critical for real-world implementation. When tested in a 2 M ZnSO_4_/0.2 M MnSO_4_ electrolyte with a cathodic mass loading of >9 mg cm^−2^ (active material >65 mg), the modified cathode delivers 401.8 mAh g^−1^ of initial capacity at 0.2 C with 93.0% capacity retention over 70 cycles (Fig. S46), representing a 6-fold scalability improvement compared with coin-cell configurations. This performance persistence becomes particularly pronounced in extended operational testing, in which Zn–MnO_2_@BiO systems maintain >20 mAh of capacity after 60 days of continuous operation (Fig. 6b). GCD curves (Fig. 6c) further corroborate the ability of the MnO_2_@BiO cathode to sustain voltage plateaus, indicative of stabilized solid-phase conversion mechanisms. Remarkably, even under extreme mass-loading conditions (16 mg cm^−2^, total active material >120 mg), the battery retains 72.7% capacity over 110 days (equivalent to 350 cycles) (Fig. 6d) and the energy density reaches 284.9 Wh kg^−1^. This performance superiority stems from the regulation feature of the SRL, enabling direct translation from coin cells to pouch configurations with high efficiency retention. Benchmarking against state-of-the-art zinc battery systems reveals the MnO_2_@BiO cathode’s unparalleled combination of cell capacity (23.1 mAh) and calendar longevity (Fig. 6e and Table S6). This performance consistency extends across production batches, as evidenced by capacity deviations of <7% in three independent cell replicates within 60 days (Fig. S47), and is imperative for its commercialization and practical application. The demonstrated electrochemical robustness positions this interfacial engineering strategy as a universal platform for developing grid-scale aqueous energy-storage solutions.

Furthermore, this interfacial engineering strategy exhibits broad applicability, as evidenced by other MnO_2_ polymorphs (e.g. ε-MnO_2_). The ε-MnO_2_ modified with Bi_2_O_3_ (denoted as ε-MnO_2_@BiO) achieves 26.6 mAh of capacity at 0.5 C with 80% retention after 400 cycles and, maintaining 70% capacity through 2700 cycles at 5 C (Fig. S48), the energy density reaches 197.6 Wh kg^−1^. Practical validation through pouch-cell prototypes (55 mg cm^−2^ of mass loading) confirms technological maturity, achieving 301 mAh of initial capacity with 87% retention after 190 cycles (Fig. 6f and g). These advancements establish a materials design paradigm in which interfacial stabilization enables the simultaneous scaling of battery energy density and cycle durability. The demonstrated performance metrics directly address critical needs in grid-scale storage and electric mobility applications, while providing a generalizable framework for developing next-generation aqueous battery systems.

## CONCLUSION

In summary, we propose a size-controlled solid–solid conversion strategy and identify multifunctional SRLs by using DFT theoretical computations. Among the candidates, Bi_2_O_3_ exhibits the strongest adsorption energy for Zn and the highest lattice compatibility with MnO_2_. By employing an epitaxial crystal growth method, we successfully coated a 2-nm-thick Bi_2_O_3_ layer onto the surface of MnO_2_, forming MnO_2_@BiO. This material achieves exceptional cycling stability and high specific capacity under high mass loading and low current density, making it suitable for grid-scale energy storage. The MnO_2_@BiO composite mitigates Mn dissolution during the initial discharge of the cathode, preventing the rapid collapse of the Mn-species structure and dynamically regulating the Mn^2+^/H^+^ concentration in the electrolyte. Meanwhile, strong adsorption with Zn promotes the uniform nucleation and deposition growth of ZHS and ZMO. Under the synergistic effects of multiple factors, the SRL enables the MnO_2_ cathode to achieve the nanoscale solid-phase conversion of ZHS–ZMO over extended cycles, ensuring long-term cycling stability. This approach offers new insights for the design of electrode materials for battery systems applicable to deposition–dissolution mechanisms, potentially accelerating the commercialization process of large-scale aqueous electrochemical energy-storage devices.

## METHODS

### Materials

*β*-MnO_2_ (referred to as MnO_2_) was synthesized via a convenient hydrothermal method, as previously reported [46]. Then, 0.211 g of MnSO_4_·H_2_O (Aladdin, 99%) and 0.285 g of (NH_4_)_2_S_2_O_8_ (Aladdin, 99.99%) were dissolved in 25 mL of deionized water at room temperature. After stirring for 30 min, the homogeneous solution was transferred to a 45-mL stainless-steel autoclave with a polytetrafluoroethylene (PTFE) liner, then heated to 140°C for 12 h. The precipitate was washed with deionized water and ethanol several times and dried at 60°C overnight.

*β*-MnO_2_@BiO (referred to as MnO_2_@BiO) was synthesized by mixing and grinding the above-obtained *β*-MnO_2_ and Bi (NO_3_)_3_·5H_2_O (Alfa Aesar, 98%) at a mass ratio of 97:3, followed by calcination in air at 500°C for 2 h at a heating rate of 5°C min^−1^.

### Electrochemical measurement

Galvanostatic charge/discharge testing of Zn–Mn full cells was conducted under a constant-current protocol by using a Neware battery tester within a voltage range of 0.8–1.9 V. CV curves were measured through a Biologic-VMP3 electrochemical workstation under a voltage range of 0.8–1.9 V at a scan rate of 0.5 mV s^−1^. Electrochemical impedance spectroscopy measurements were performed on a Biologic-VMP3 electrochemical workstation with an AC voltage for different cycle numbers in the frequency range of 10 mHz to 1 MHz. All electrochemical tests were performed at room temperature and the capacity measurements were based on the mass of the active material in the cathode. EQCM testing was performed by coupling the QSense Initiator (QCM instrument) with the Biologic VSP-3e (electrochemical workstation). More details are available in the Supplementary information.

### Computational details

The projection-enhanced plane wave (PAW) method [47] in the VASP package [47,48] was used under the GGA’s Perdew–Burke–Ernzerhof [38] exchange-correlation generalization of the GGA [49] (The first-principles study used the VASP package [48] of the Projector-enhanced PAW method [50].) The DFT-D3 method [51] was used to calculate the van der Waals interactions. The kinetic truncation energy was set to 520 eV for all calculations. Also, 2 × 2 × 1 supercells of Bi_2_O_3_ and 5 × 2 × 1 supercells of MnO_2_ were fully relaxed and their geometries were optimized. The energy-convergence criterion was set to 10^−5^ eV and the force-convergence criterion was set to 0.05 eV Å^−1^ for the structure optimization process. A 2 × 2 × 1 Monkhorst–Pack grid was used for K-point sampling, while a 3 × 3 × 1 K-point grid with a higher density was used for the calculation of the electronic structure properties. At the same time, a 20-Å vacuum layer was set up in the 2D vertical direction to avoid interactions between the periodic structures.

The adsorption energy (*E*_ads_) was determined by using:


(7) \begin{eqnarray*}
{E}_{{\mathrm{ads}}} = {E}_{{\mathrm{total}}} - {E}_{{\mathrm{slab}}} - {E}_{{\mathrm{gas}}},
\end{eqnarray*}

where the total energy of the gas adsorption on the slab is represented by *E*_total_, while *E*_slab_ and *E*_gas_ represent the energies of the materials and intermediate, respectively.

## Supplementary Material

nwag010_Supplemental_File
