# Supplementary material for: Revitalizing nanoscale solid–solid conversion enables ultrastable aqueous batteries
Source: Natl Sci Rev. 2026 Jan 13;13(5):nwag010. doi: 10.1093/nsr/nwag010 (PMC12997404; doi:10.1093/nsr/nwag010)
Supplement: nwag010_Supplemental_File [file nwag010_supplemental_file.pdf]

Supplementary Information

Revitalizing nanoscale solid-solid conversion enables ultrastable aqueous batteries

Zhixing Sun[1]#, Mei Han[2,3]#, Yuchun Liu[1]#, Hang Wang[1], Xingwu Zhai[1], Liang Wu[1], Zhuohui Zhang[1], Jian Zhi[2]*, Pu Chen[3,4]*, Min Zhou[1]*

1. Hefei National Laboratory for Physical Sciences at the Microscale, School of Chemistry and Materials Science, University of Science and Technology of China, Hefei, 230026 China

2. State Key Laboratory of High Performance Ceramics, Shanghai Institute of Ceramics, Chinese Academy of Sciences, Shanghai, 200050, China

3. Department of Chemical Engineering and Waterloo Institute of Nanotechnology, University of Waterloo, N2L 3G1, Canada

4. School of Chemical and Biomolecular Engineering, College of Engineering, Eastern Institute of Technology, Ningbo, Zhejiang, 315200, China

#These authors contributed equally to this work.

*Email: jzhi@mail.sic.ac.cn, p4chen@uwaterloo.ca, mzchem@ustc.edu.cn

Materials and methods

Materials

β-MnO_2@AlO and β-MnO_2@TiO were synthesized in essentially the same way as β-MnO_2@BiO. β-MnO_2@AlO was synthesized with a 3% mass ratio of Al $(NO_3)_3 \cdot 9H_2O$ (Aladdin, 99%), and β-MnO_2@TiO was synthesized with a 3% mass ratio of TBOT (Sinopharm, 98%).

ε-MnO_2 was synthesized by co-precipitation followed by calcination. Specifically, 200 mL 1.05 M Na_2CO_3 solution and 200 mL 1.00 M $MnSO_4$ solution were gradually added to 30 mL of ethanol under continuous stirring, followed by stirring at 600 rpm for 2 h, and then standing for 2 h. Afterwards, the precursor ($MnCO_3$) was washed several times with DI water and ethanol, respectively. The washed sample was dried under vacuum at 60℃ overnight. The dried precursor was calcined in a muffle furnace at 410℃ for 8 hours to obtain ε-MnO_2. ε-MnO_2@BiO was β-MnO_2@BiO in the same way as β-MnO_2@BiO.

Characterizations

The morphology and structure of the materials or cathode were investigated by scanning electron microscope (SEM, ZEISS Gemini SEM 360& FEI Quanta FEG 250) and X-ray diffraction (XRD, Rigaku SmartLab SE) with Cu-Kα radiation (λ = 1.54178 Å). The chemical state of the materials was determined by X-ray photoelectron spectrometer (XPS, Thermo Scientific ESCALAB 250Xi) using Al-Kα source (hν=1486.6 eV), and the binding energy was corrected by referencing C 1s to 284.8 eV. The in-situ X-ray diffraction patterns obtained during the charge/discharge process were recorded with Rigaku TTR III. Phase contrast diffraction images and energy dispersive spectrometer analysis were captured by High-resolution transmission electron microscopy (HRTEM, JEOL JEM-2100 Plus) at the acceleration voltage of 200 KV. High-angle annular dark-field scanning transmission electron microscopy (HAADF-STEM) and electron energy loss spectrum (EELS)

measurements were performed on aberration-corrected transmission electron microscope (ACTEM, JEOL JEM-ARM200F). The elemental content of electrodes was obtained by Inductively Coupled Plasma Optic Emission Spectrometer (ICP-OES, Pekin Elmer Avio 220 MAX), where the electrodes were first dissolved in concentrated hydrochloric acid, diluted and tested. Synchrotron radiation soft X-ray absorption spectroscopy (s-XAS) of Mn K-edge was performed at the BL12B-b beamline of the National Synchrotron Radiation Laboratory (NSRL) in Hefei.

Cathode fabrication and battery assembly

Cathode fabrication

A slurry containing 80 wt.% active material (e.g., β-MnO_2), 5 wt.% acetylene black (AB), 5 wt.% TIMREX KS-6 and 10 wt.% polyvinylidene difluoride (PVDF) was prepared in 1-Methyl-2-pyrrolidinone (NMP) solution. This slurry was uniformly coated onto the conductive polyethylene (PE) film by a doctor blade technique. The cathode sheets were then vacuum-dried overnight at 60 ℃ and used for coin-type cells with a thickness of 400 μm and for large cells with a thickness of 650 μm.

The composition of cathode sheet for pouch cell is 78 wt.% ε-MnO_2, 8 wt.% AB, 7 wt.% TIMREX KS-6 and 7 wt.% polytetrafluoroethylene (PTFE) on an aluminum foil as a current collector with the active material mass loading around 55 mg cm^{-2}.

Coin-type battery assembly

CR2025 coin-type battery was utilized for electrochemical tests. The coin-type cell was assembled with a cathode (diameter:12 mm, mass loading of active material: 9.0±0.2 mg cm^{-2}), a single-layered absorbed glass mat (AGM, NSG Corporation, diameter: 16 mm, thickness: 0.4 mm, mass: 25.0±0.1 mg) as a separator, and zinc foil (Rotometals, diameter: 12 mm, thickness: 0.02 mm, mass: 15.0±0.1 mg) as an anode. 120 μl 2 M $ZnSO_4$/0.2 M $MnSO_4$ solution with pH=4.0±0.1 was used as electrolyte. The calendaring pressure of the coin-type battery is about 800 psi and hold for 5 seconds.

Large (iron-plate) battery assembly

Large batteries or iron plate batteries were assembled with a cathode sheet (2.5 cm×3 cm), double-layered AGM (4 cm×4 cm) as separator between cathode and anode, and zinc foil (3 cm×3 cm, thickness: 0.03 mm) as anode, and tightened with screws. The electrolyte amount for large battery is approximately 1.5 mL.

Pouch-type battery assembly

Pouch cells were assembled with a cathode piece (6 cm×6 cm), a single-layered AGM (7 cm×8 cm×0.4 mm) and a zinc foil (6 cm×6 cm×0.03 mm). The components were sequentially layered—cathode, separator, anode—and fixed with adhesive tape. The cut tabs are connected to the cathode and anode respectively using an ultrasonic welding machine, and the welded points are insulated with high-temperature tape to fabricate a standard cell. This assembly was then encased within a 9 cm×10 cm aluminum-plastic film, and an appropriate amount of 2 M $ZnSO_4$/0.2 M $MnSO_4$ electrolyte was injected into the cell under vacuum. The cell was pressurized for 10 minutes before being vacuum-sealed to finalize the pouch cell fabrication.

Electrochemical measurement

Electrochemical quartz crystal microbalance (EQCM)

Cathode materials, acetylene black (AB), and carboxymethylcellulose (CMC) were mixed in a mass ratio of 80:15:5 with deionized water to form an extremely dilute slurry, then applied on the Au-coated quartz crystal. The EQCM cell was assembled using Zn foil as the anode and 2 M $ZnSO_4$/0.2 M $MnSO_4$ as the electrolyte. The EQCM response is related to the mass change of the electrode due to the interaction of ions and/or solvent molecules during electron transfer, according to the following Sauerbrey equation (Eq (1)):

$$\Delta \mathrm{f} = -C_f \cdot \Delta m \tag{1}$$

where Δf and Δm represent frequency and mass change, respectively. C_f is the sensitivity factor for the crystal (56.6 Hz μg^{-1} cm^2). By applying a current or voltage

to the EQCM cell, the apparent molar mass of the deposited or adsorbed material related to charge transfer can be calculated by Faraday's law in the Eq (2):

$$\frac{\Delta m}{\Delta Q} = \frac{M_w}{n \cdot F} \qquad (2)$$

where ΔQ is the charge passed through the electrode (C), M_w is the molar mass of the sedimentary species (g mol^{-1}), n is the number of transferred electrons, and F is the Faraday constant (96485 C mol^{-1}).

Supplementary Fig.s

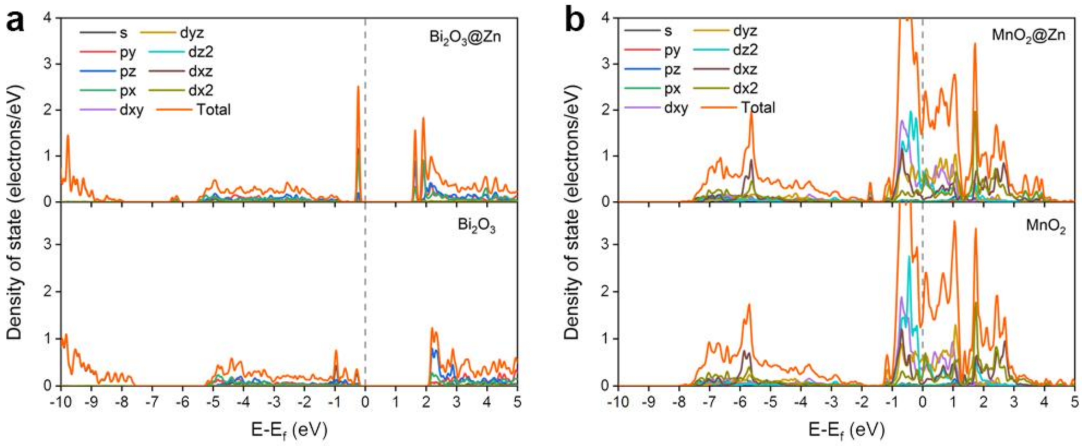

Supplementary Fig. 1: DOS before and after Zn adsorption by **a** Bi_2O_3 and **b** MnO_2.

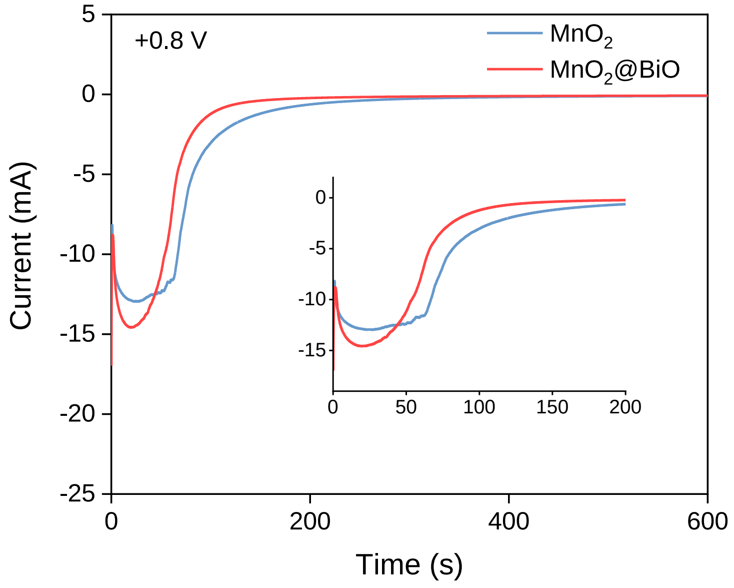

Supplementary Fig. 2: Chronoamperometry (CA) curves of MnO_2@BiO and MnO_2 at +0.8V overpotential. The test was conducted in a two-electrode system, with the positive electrodes being MnO_2@BiO and MnO_2, and the negative electrode being Zn foil. The electrolyte is 2M $ZnSO_4$+0.2M $MnSO_4$ aqueous solution, and the set voltage is +0.8V vs Zn/Zn^{2+}.

In the i-t curves shown in **Supplementary Fig. 2**, the evolution of the current with time indicates nucleation behavior. After an initial rise followed by a decay, a current maximum (i_{max}) is reached at the peak time (t_{max}). A larger i_{max} indicates a higher nucleation density, a shorter t_{max} reflects faster nucleation. Moreover, the steep slope of the rising branch ($(di/dt)_{rising} \propto N$)signals a high density of active sites, and the rapid current drop marks the end of the nucleation stage. Evidently, MnO_2@BiO exhibits both faster and denser nucleation events, corroborating that the Bi_2O_3 reduces the nucleation barrier and shortens the nucleation period.

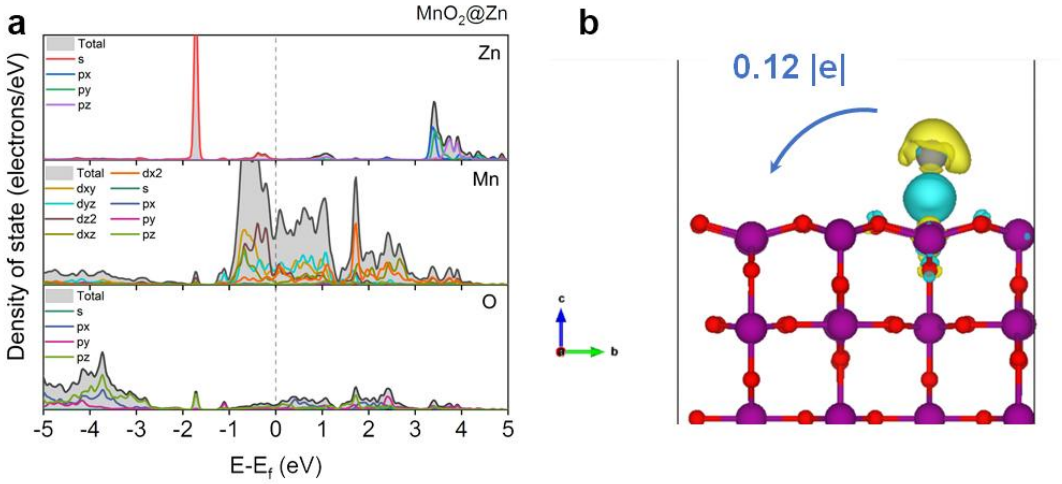

Supplementary Fig. 3: a DOS curves of MnO_2@Zn showing the hybridization between Zn^{2+} and Mn/O atoms in MnO_2. **b** The charge density difference mapping showing the charge transfer between Zn^{2+} and MnO_2.

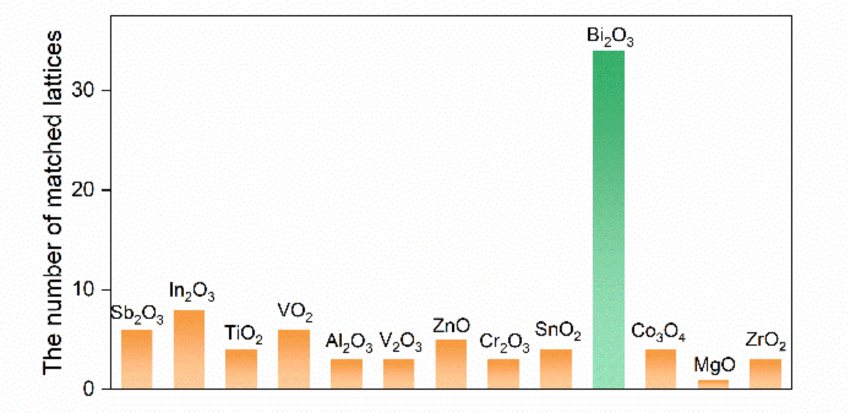

Supplementary Fig. 4: Comparison of the number of crystal facets with less than 5% mismatch between each oxide phase and MnO_2 (001) facet. (Calculation method: take MnO_2(001) crystal facet spacing d=2.865Å as a reference, and count the number of crystal facets in all crystal facets of each oxide that have a difference of less than 5% in the mismatch rate of 1x, 1.5x, and 2x of the spacing of the crystal planes of (001).)

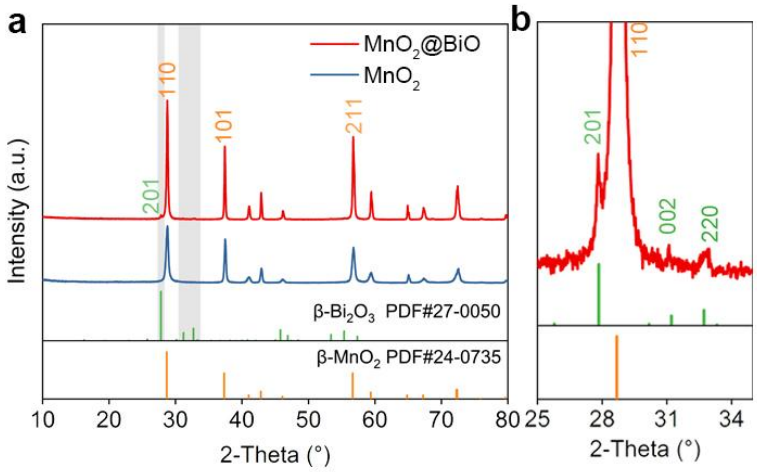

Supplementary Fig. 5: a XRD patterns of MnO_2@BiO and MnO_2 **b** The magnified XRD diffraction pattern at the location marked by the red dashed line in **a**.

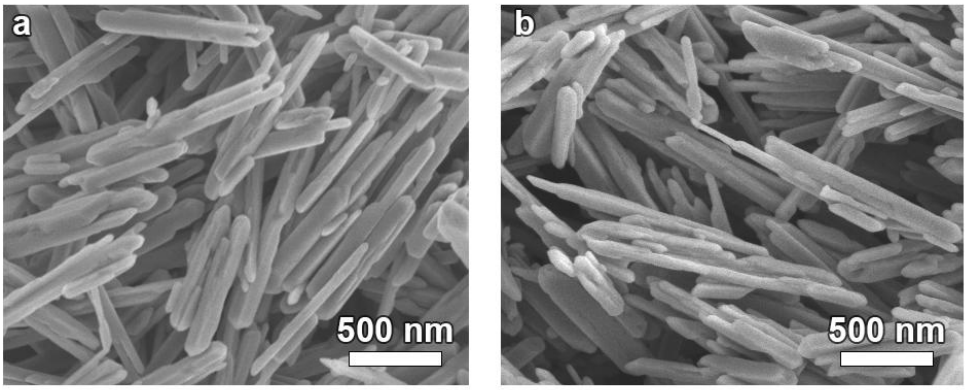

Supplementary Fig. 6: SEM images of **a** MnO_2 and **b** MnO_2@BiO.

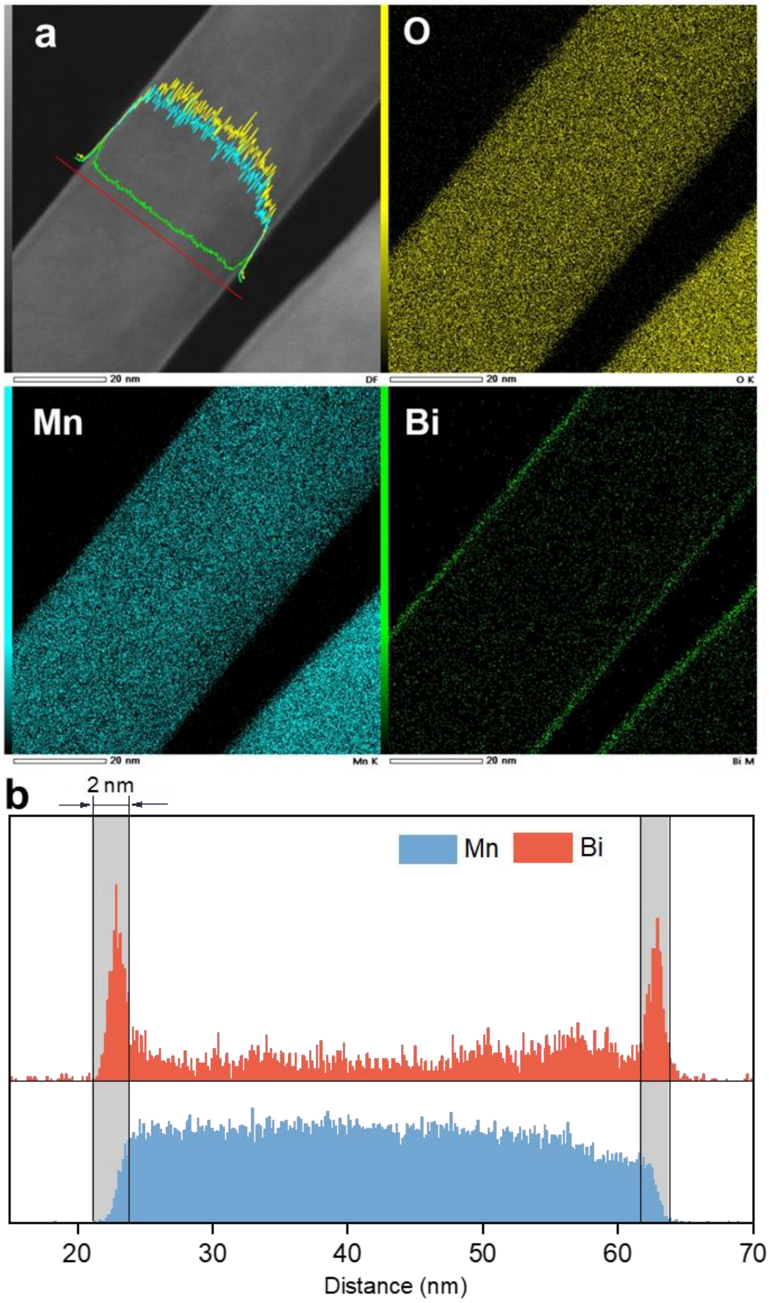

Supplementary Fig. 7: a High-angle annular dark field-scanning transmission electron Microscopy (HAADF-STEM) image of MnO_2@BiO and EDS line scanning and corresponding EDS elemental distribution maps of O, Mn and Bi, **b** corresponding EDS line scanning of Mn and Bi.

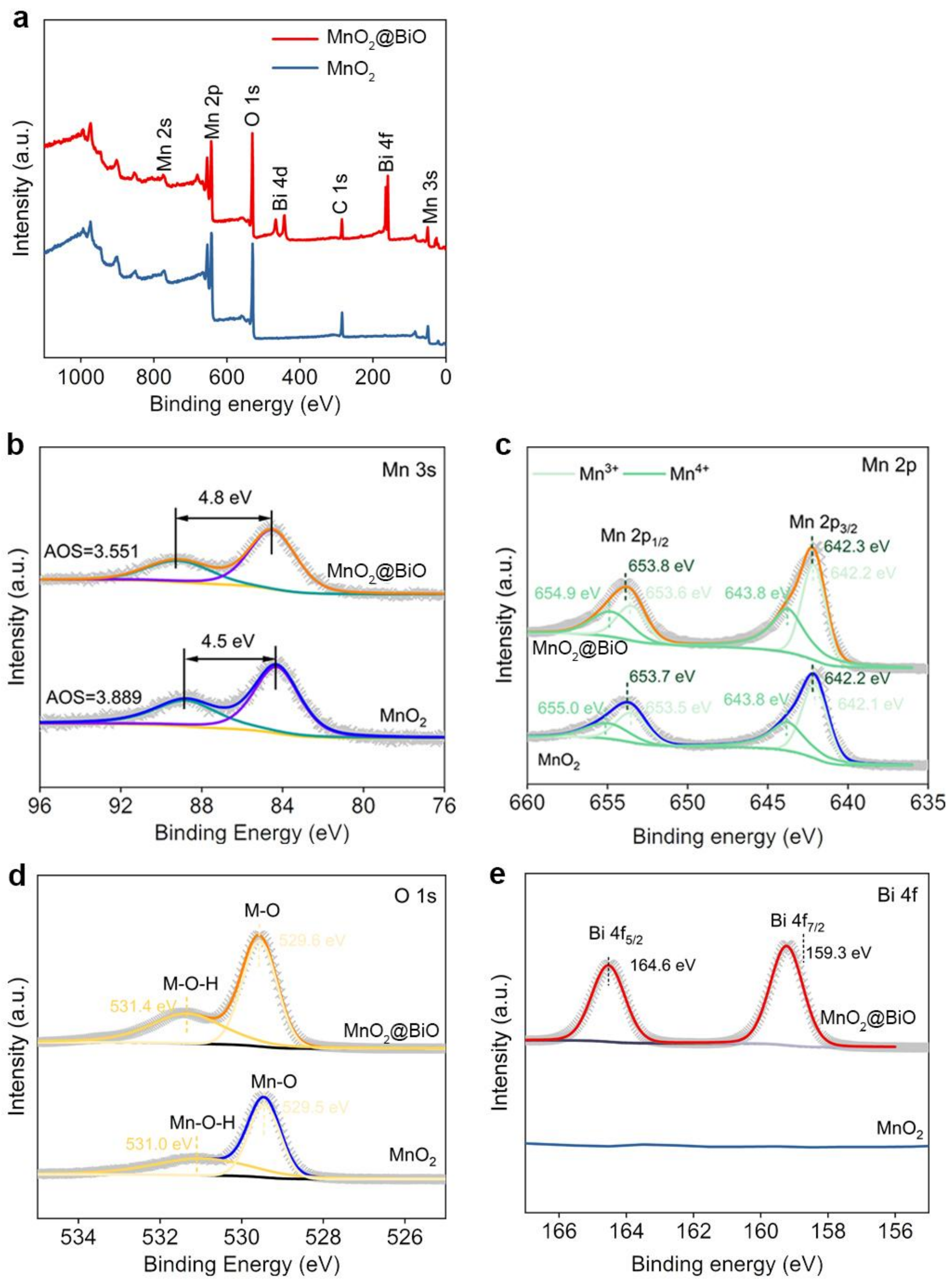

Supplementary Fig. 8: XPS of MnO_2@BiO and MnO_2 **a** full Spectrum, **b** Mn 3s, **c** Mn 2p, **d** O 1s and **e** Bi 4f.

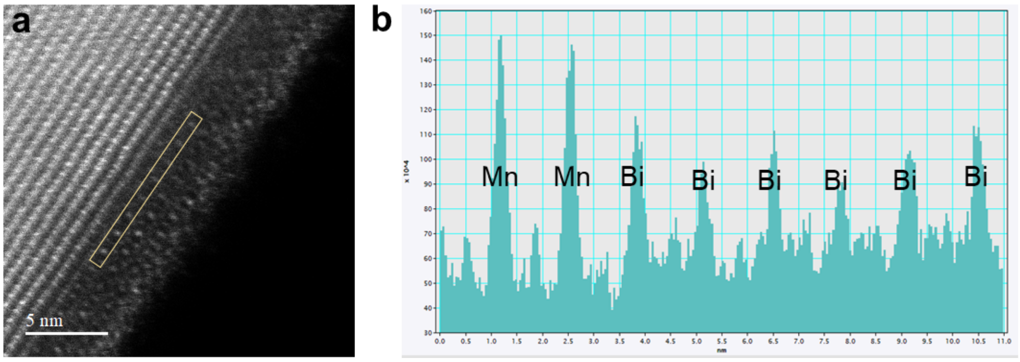

Supplementary Fig. 9: AC-HAADF-STEM image of **a** MnO_2@BiO (3wt%), and **b** grayscale intensity spectrum in the vertical direction of the crystal plane (the marked part in **a**).

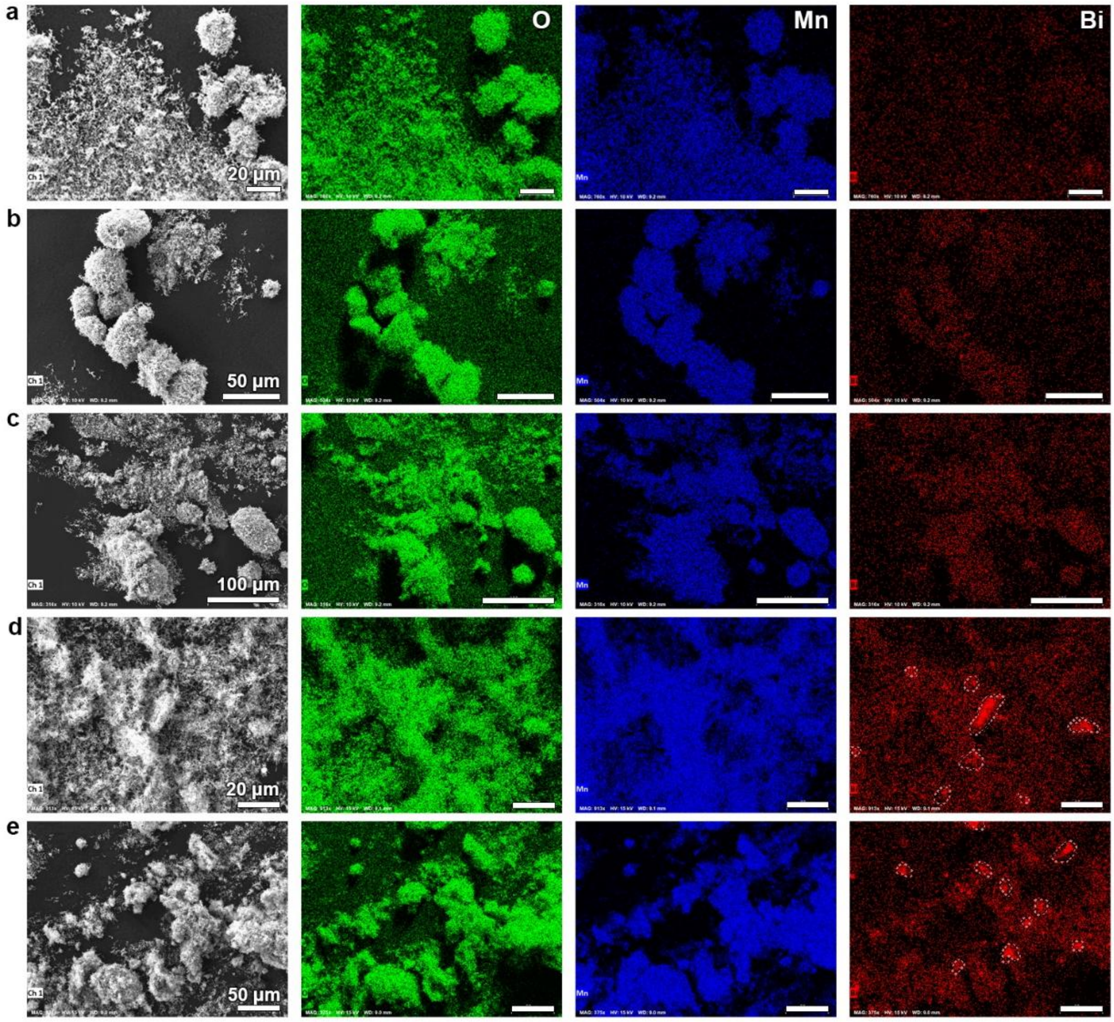

Supplementary Fig. 10: SEM images and EDS elemental distribution maps of Mn, O and Bi of MnO_2@BiO with **a** 1 wt%Bi$(NO_3)_3$, **b** 3 wt%Bi$(NO_3)_3$, **c** 5 wt%Bi$(NO_3)_3$, **d** 10 wt%Bi$(NO_3)_3$ and **e** 15 wt%Bi$(NO_3)_3$. The dotted line circles the accumulation of bismuth trioxide.

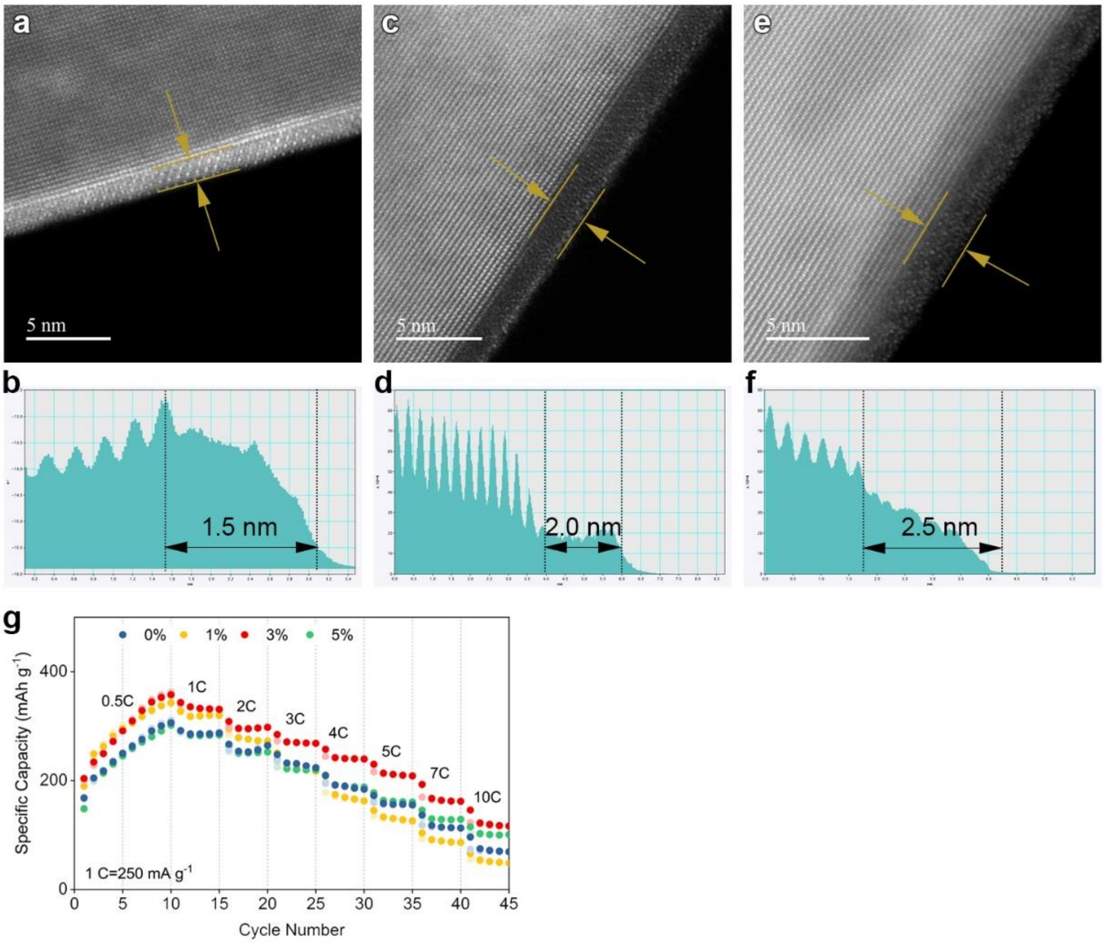

Supplementary Fig. 11: HAADF-STEM images of MnO_2@BiO with **a, b** 1 wt% $Bi(NO_3)_3$, **c, d** 3 wt%$Bi(NO_3)_3$, **e, f** 5 wt%$Bi(NO_3)_3$ and **g** corresponding Zn-Mn batteries performance, electrolyte: 2 M $ZnSO_4$/0.2 M $MnSO_4$, anode: Zn foil.

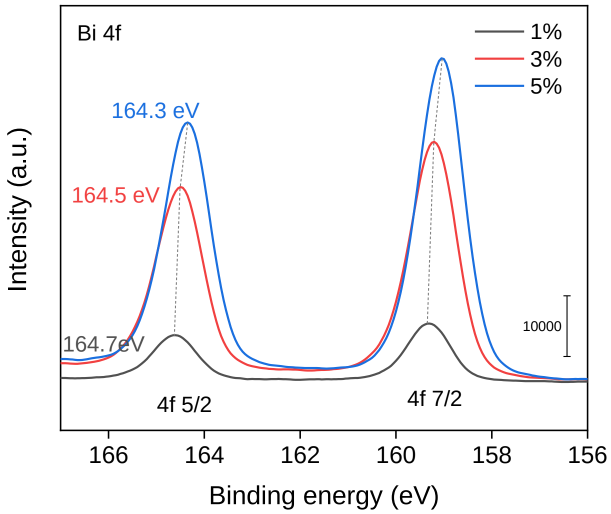

Supplementary Fig. 12: High-resolution Bi 4f XPS spectra of the MnO_2@BiO with 1wt%, 3wt% and 5wt% Bi.

Given identical calcination time during synthesis and assuming negligible thickness-dependent effects on diffusion coefficients, the characteristic diffusion length of Mn in Bi_2O_3 is presumed to be similar across samples. XPS analysis within the probe depth (~3-10 nm) revealed a gradual positive shift in Bi 4f binding energy as the Bi_2O_3 layer thickness decreased from 2.5 nm (5 wt% Bi) to 1.5 nm (1 wt% Bi). This phenomenon arises from diffusion spatial distribution effects, at comparable diffusion lengths, thinner Bi_2O_3 layers (1.5 nm) exhibit higher Mn concentrations near the surface due to shorter diffusion distances. Following Mn incorporation into the Bi_2O_3 lattice, Mn-O-Bi bonding induces localized charge redistribution (reducing electron density around Bi and increasing it around Mn). The binding energy shift intensity directly reflects the proportion of Bi atoms affected by Mn within the XPS detection depth. The shift is more pronounced in thinner samples, indicating a higher number of Bi atoms in modified coordination environments (Mn-O-Bi structures) near the surface.

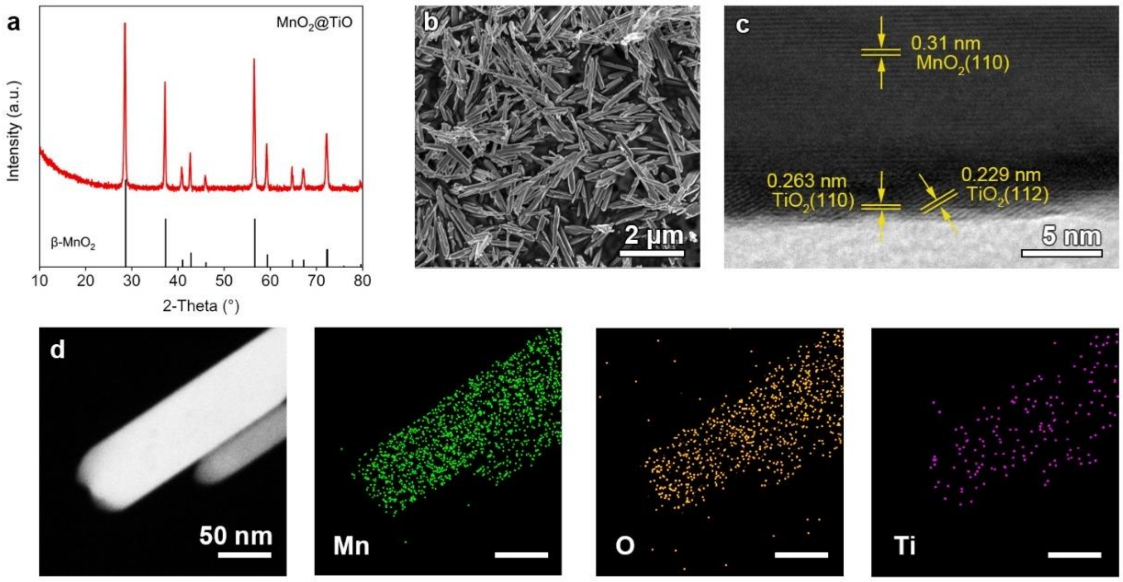

Supplementary Fig. 13: a XRD patterns of MnO_2@TiO, **b** SEM images of MnO_2@TiO and **c** HRTEM images of MnO_2@TiO, **d** corresponding HAADF-STEM image and elemental distribution maps of Mn, O and Ti.

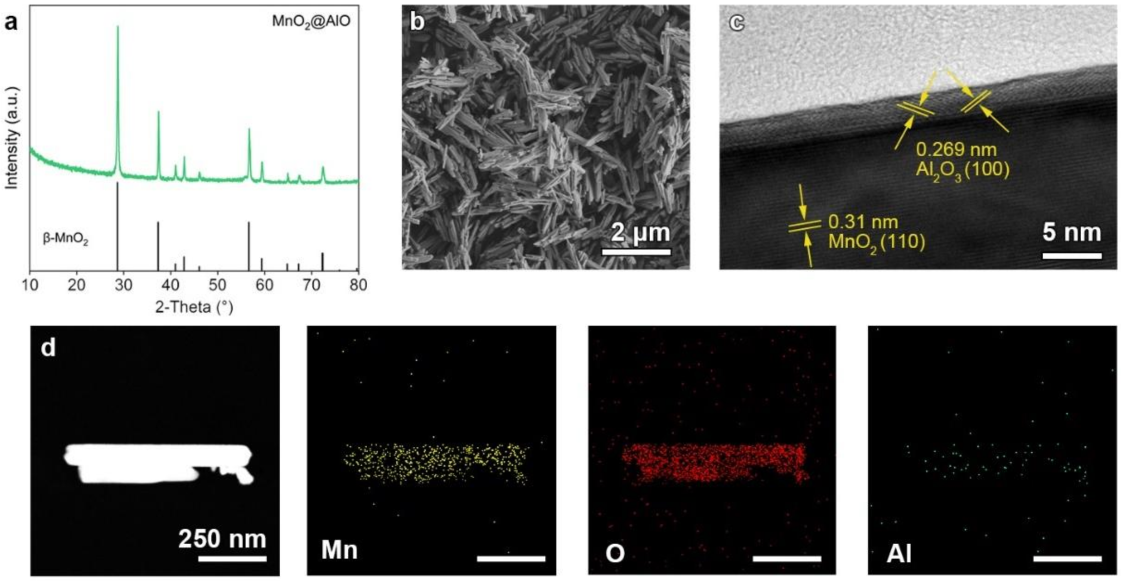

Supplementary Fig. 14: **a** XRD patterns of MnO_2@AlO, **b** SEM images of MnO_2@AlO and **c** HRTEM images of MnO_2@AlO, **d** corresponding HAADF-STEM image and elemental distribution maps of Mn, O and Al.

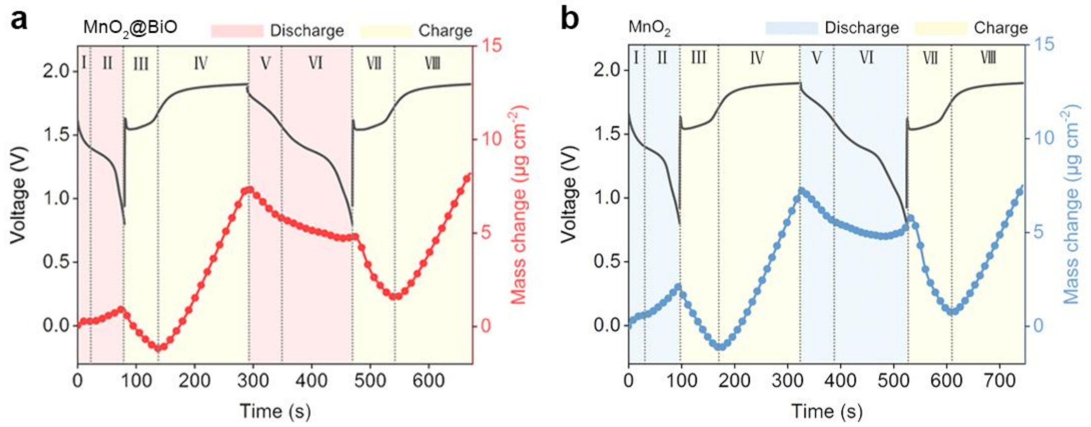

Supplementary Fig. 15: Typical the 1st and 2nd cycle galvanostatic discharge and charge profiles of Zn-Mn batteries and the corresponding mass change of the cathodes recording by Electrochemical quartz crystal microbalance (EQCM) with **a** MnO_2@BiO and **b** MnO_2 as cathode.

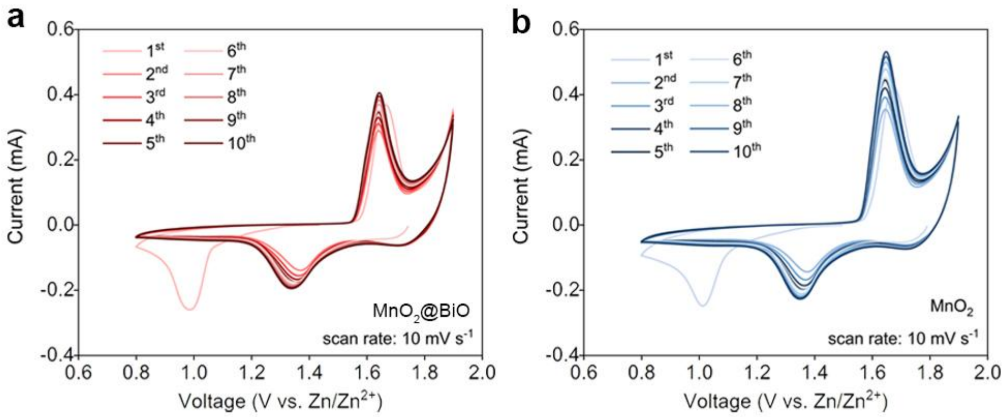

Supplementary Fig. 16: CV curves of Zn-Mn batteries with **a** MnO_2@BiO and **b** MnO_2 as cathode.

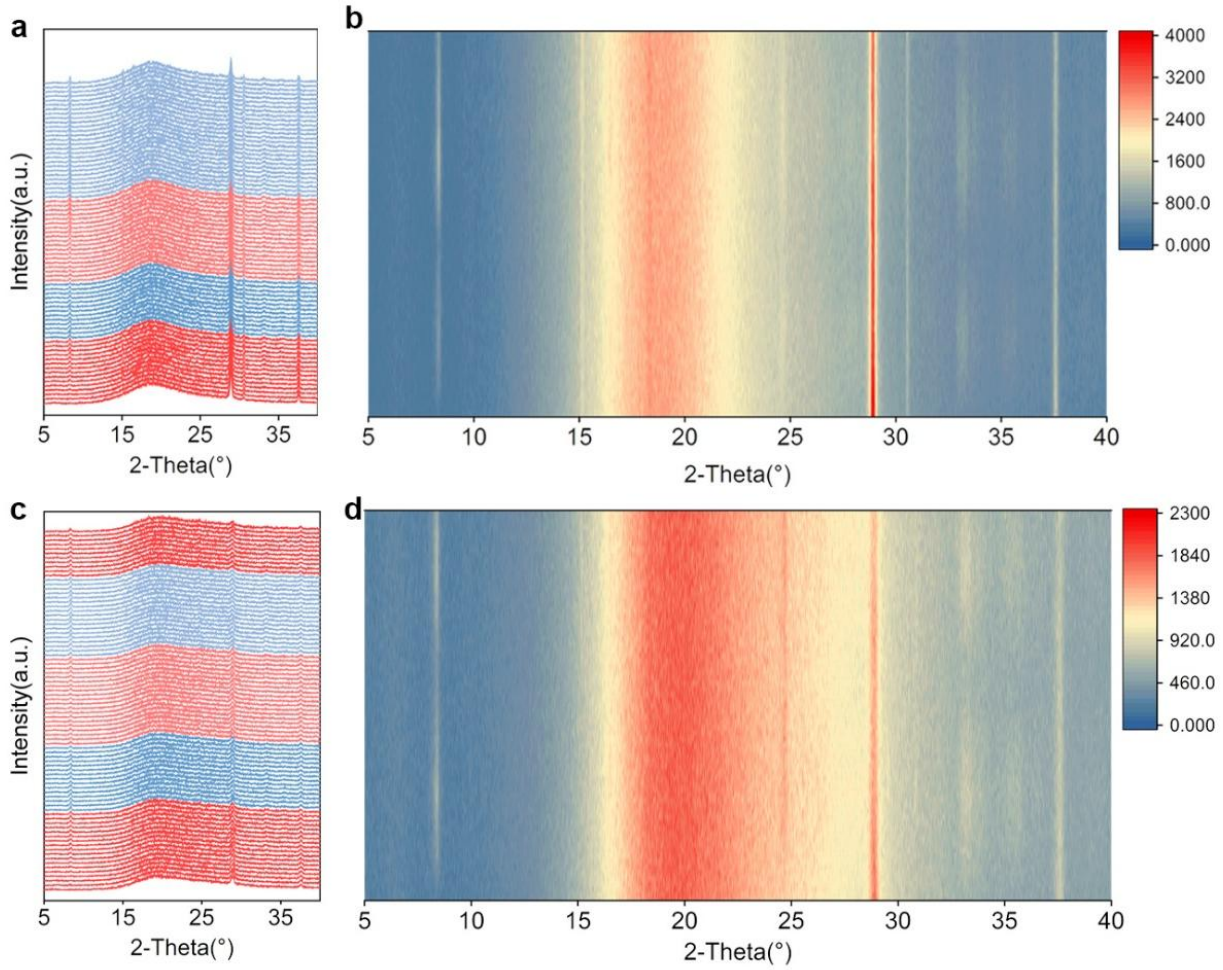

Supplementary Fig. 17: Typical the 1st and 2nd cycle in-situ XRD patterns of the **a**, **b** MnO_2@BiO and **c**, **d** MnO_2 cathodes.

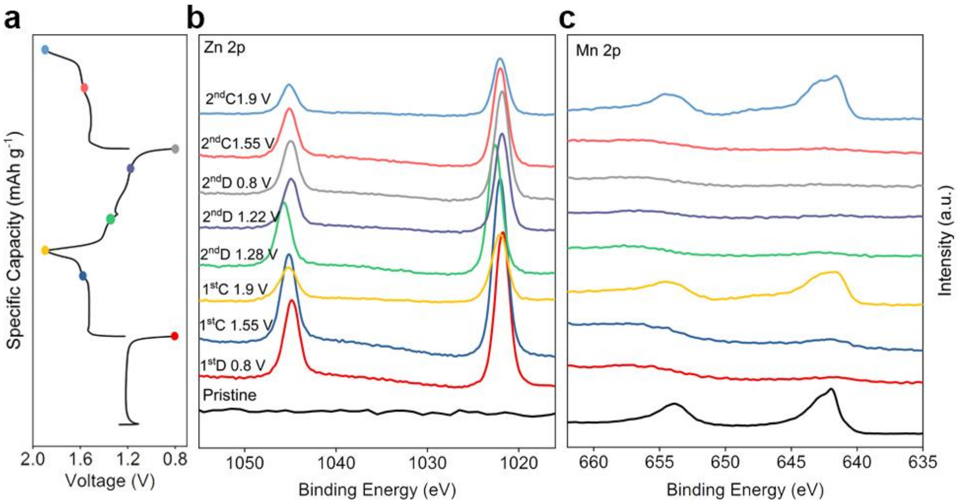

Supplementary Fig. 18: a The 1st and 2nd cycle galvanostatic discharge and charge profiles of Zn-Mn batteries with MnO_2@BiO, and corresponding high-resolution **b** Zn 2p and **c** Mn 2p XPS spectra of the MnO_2@BiO electrode at different charge and discharge cut-off voltages.

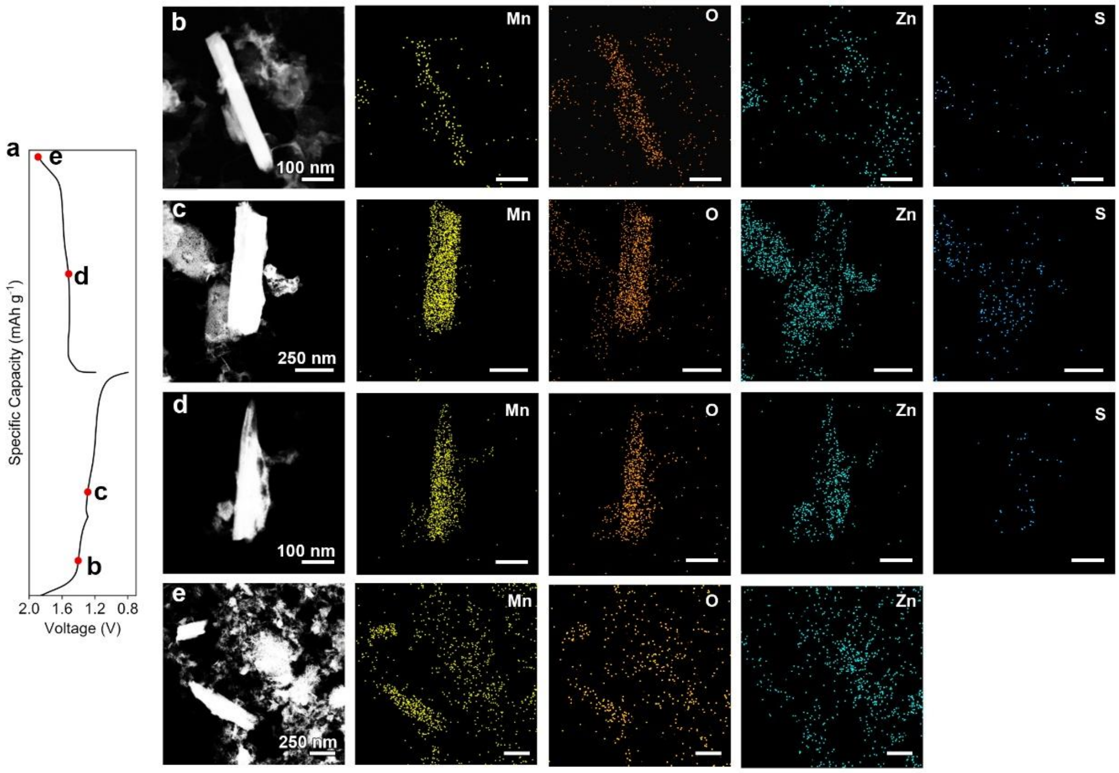

Supplementary Fig. 19: a 1st and 2nd cycle galvanostatic discharge and charge profiles. STEM-HAADF images and corresponding EDS maps of MnO_2@BiO electrodes at different discharge and charge states, including **b** 2nd discharge to 1.4 V, **c** 2nd discharge to 1.22 V, **d** 2nd charge to 1.55 V and **e** 2nd charge to 1.9 V, respectively.

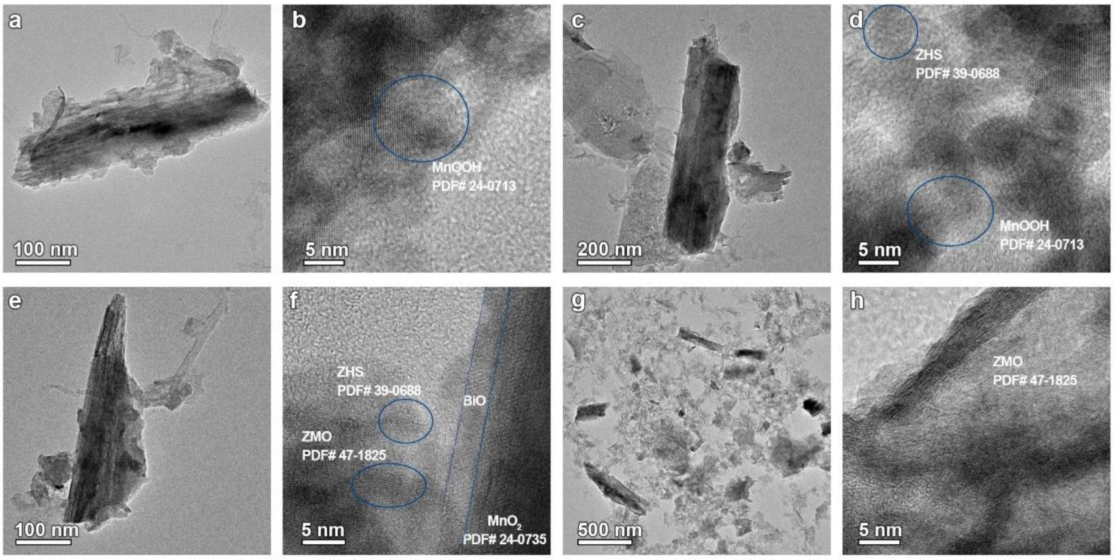

Supplementary Fig. 20: TEM and HRTEM images of MnO_2@BiO cathodes at different discharge and charge states, including **a**, **b** 2nd discharge to 1.28 V, **c**, **d** 2nd discharge to 1.22 V, **e**, **f** 2nd charge to 1.55 V and **g**, **h** 2nd charge to 1.9 V, respectively.

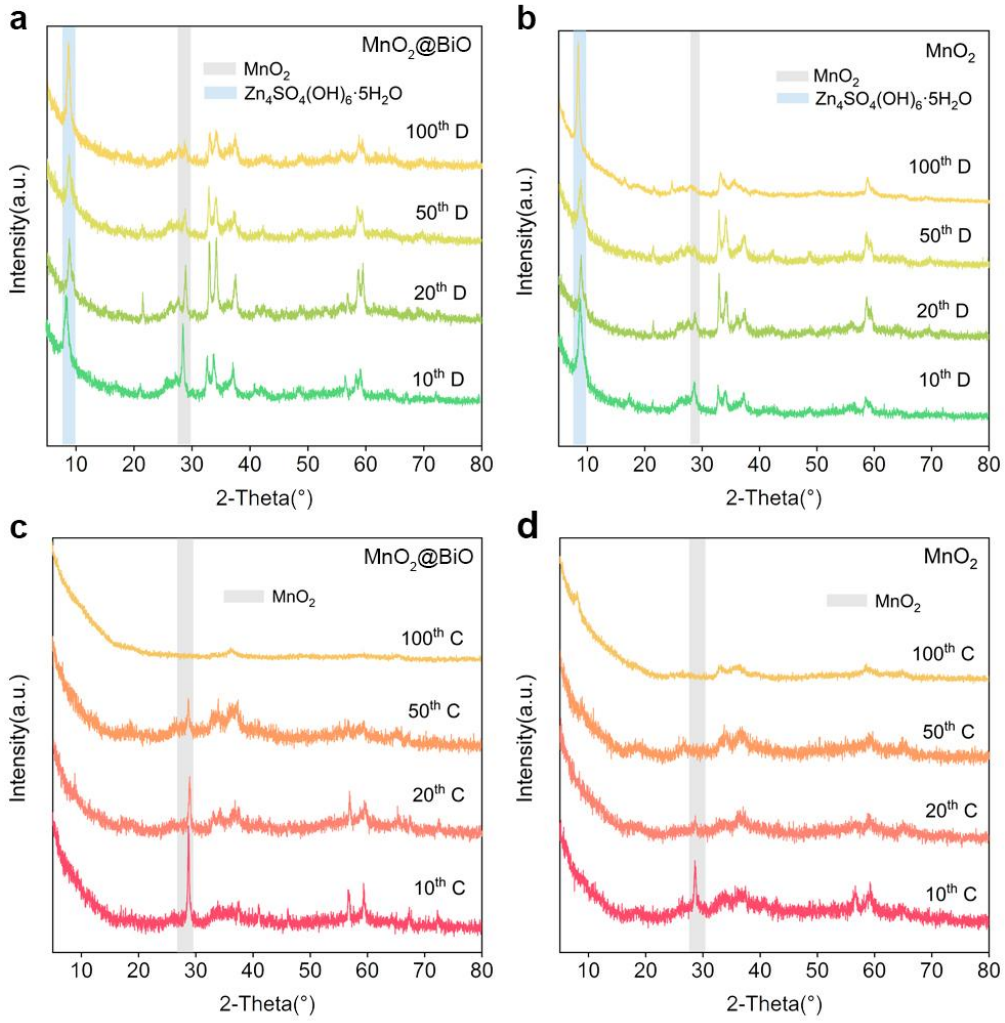

Supplementary Fig. 21: XRD patterns at fully discharged and charged after 10th, 20th, 50th and 100th of **a**, **c** MnO_2@BiO and **b**, **d** MnO_2.

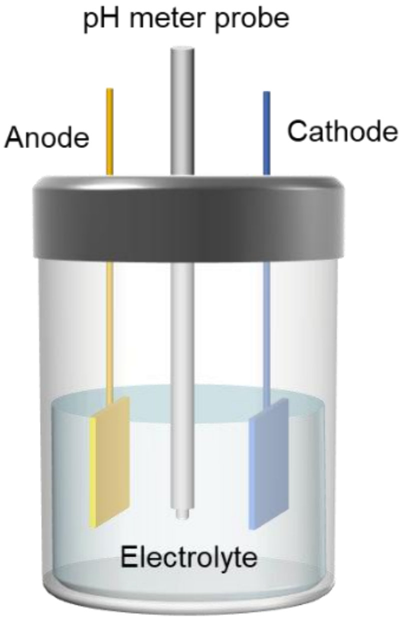

Supplementary Fig. 22: Schematic diagram of in-situ pH test two electrode beaker cell.

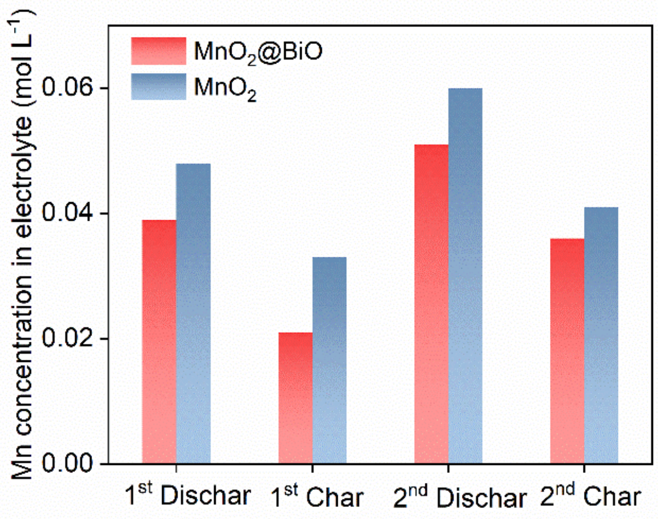

Supplementary Fig. 23: Comparison of the concentration of Mn ions in the electrolyte in the fully charged and discharged states of the 1st and 2nd cycle measured by ICP (initial electrolyte: 2 M $ZnSO_4$).

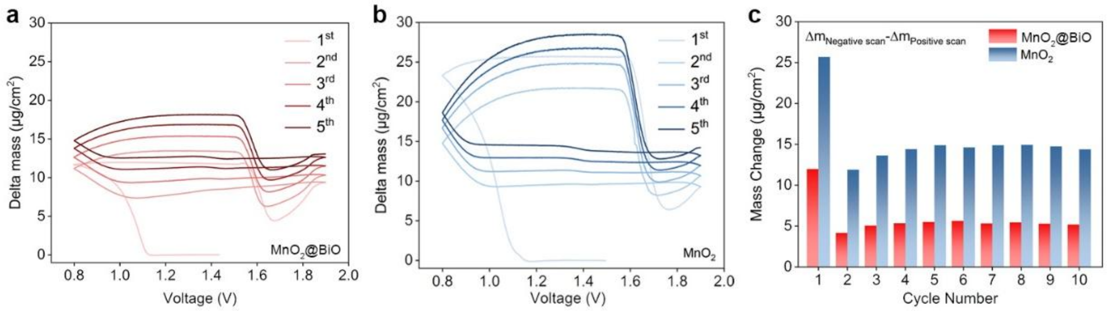

Supplementary Fig. 24: The mass change in the initial CV tests of **a** MnO_2@BiO cathode and **b** MnO_2 cathode. **c** The mass change of the cathodes during the first 10 cycles of CV scanning at 10 mV s^{-1} recording by Electrochemical quartz crystal microbalance (EQCM). The mass change per cycle was calculated as the mass change of the negatively scanned electrode minus the mass change of the positively scanned electrode.

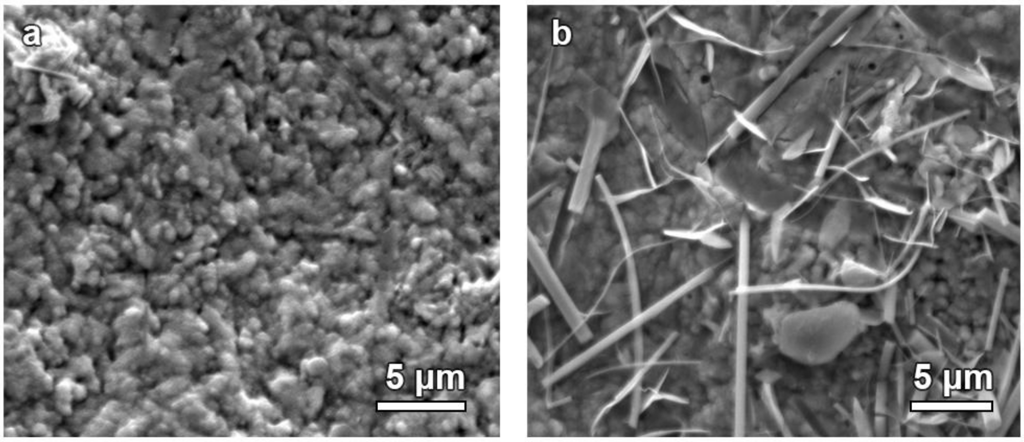

Supplementary Fig. 25: SEM images of **a** MnO_2@BiO and **b** MnO_2 at the 2nd fully charged to 1.9 V.

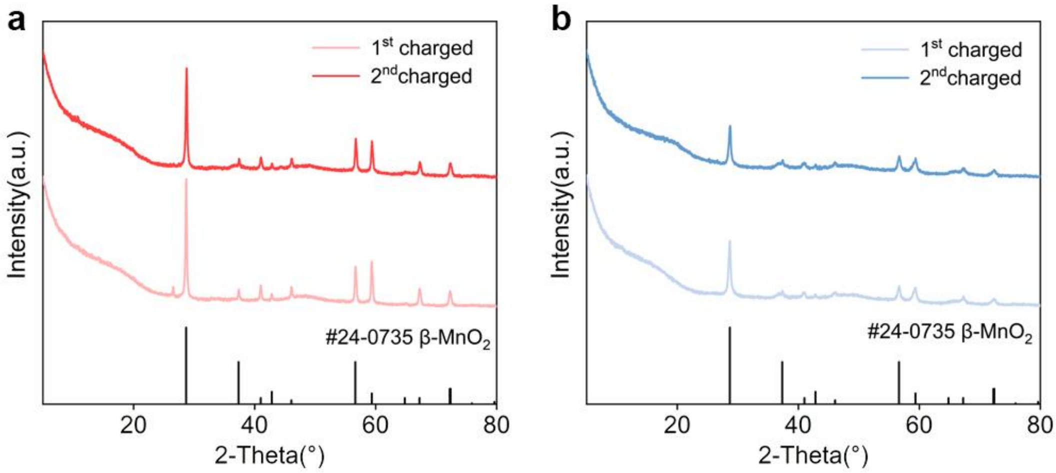

Supplementary Fig. 26: XRD patterns of **a** MnO_2@BiO and **b** MnO_2 cathodes at fully 1st and 2nd charged.

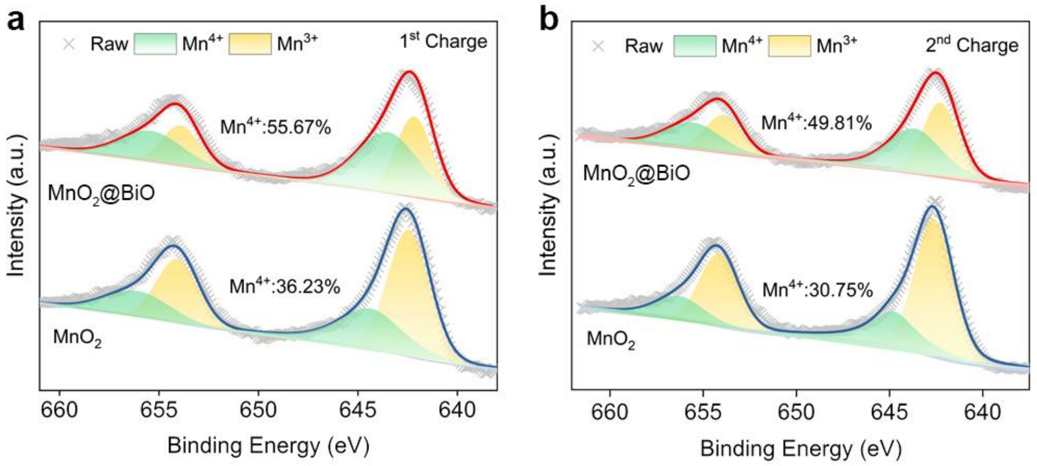

Supplementary Fig. 27: 1st and 2nd cycle high-resolution Mn 2p XPS spectra of the **a** MnO_2@BiO electrode and **b** MnO_2 at fully charge and discharge states.

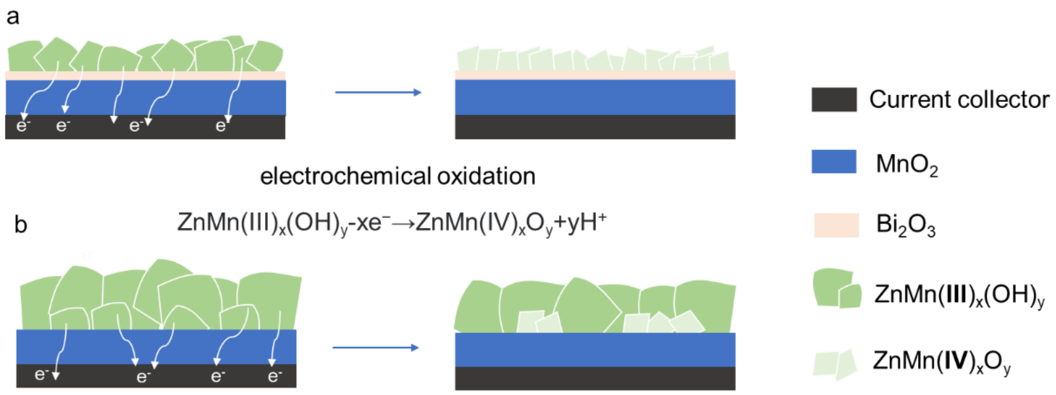

Supplementary Fig. 28: Schematic diagrams of electrochemical oxidation during charge in **a** MnO_2@BiO and **b** MnO_2 electrodes.

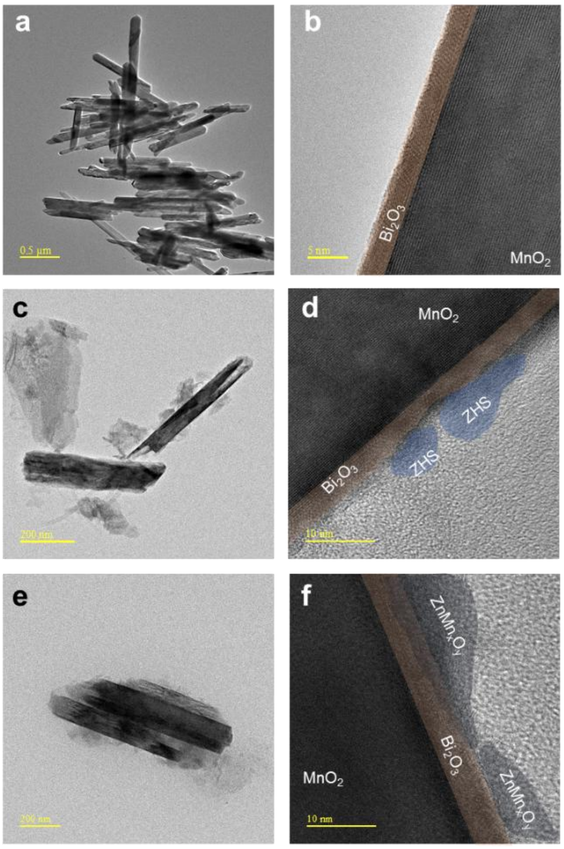

Supplementary Fig. 29: HRTEM images of MnO_2@BiO cathode. **a** pristine MnO_2@BiO and **b** enlarged image; **c** MnO_2@BiO after 20th full discharged and **d** enlarged image; **e** MnO_2@BiO after 20th full charged and **f** enlarged image.

In the pristine material (**Supplementary Fig. 29a, b**), a uniform 2 nm thick Bi_2O_3 coating on the MnO_2 surface is clearly observed. After the 20th full discharge, nanorod structures are retained (**Supplementary Fig. 29c, d**), whose surface covered by flake-shaped discharge products. High-magnification images reveals that the Bi_2O_3 coating layer and its heterointerface with the MnO_2 substrate remains discernible with minimal thickness change, although slight dissolution was evident on the Bi_2O_3 outer surface, indicated by localized concave features. The cathode after the 20th full charge also retained intact rod-like structures (MnO_2@BiO) (**Supplementary Fig. 29e, f**), where the heterointerface between MnO_2 and Bi_2O_3 remained smooth and intact, covered by the charge products on the outer surface. This interfacial integrity demonstrates that the MnO_2@BiO structure effectively tolerates the possible volumetric expansion/contraction changes of the cathode during the initial cycle reactions.

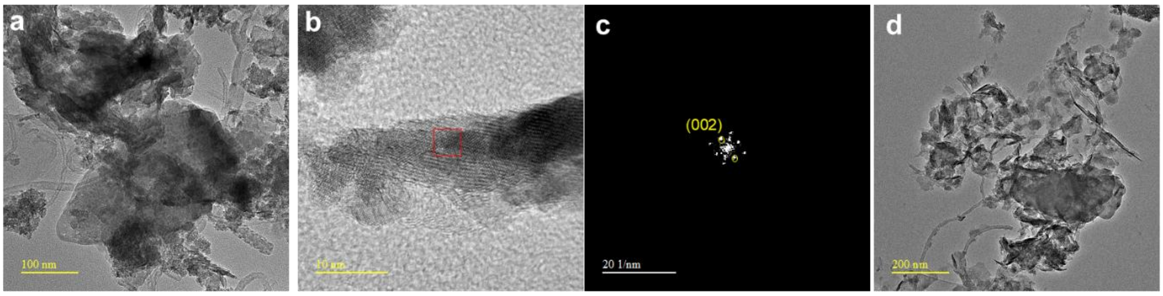

Supplementary Fig. 30: a HRTEM image of MnO_2@BiO cathode after 100th full discharged and **b** enlarged image, **c** FFT of section marked in **b**, **d** HRTEM image of MnO_2@BiO cathode after 100th full charged.

About cathodes after the 100th full discharge and full charge, the original rod-like structures had completely disappeared (**Supplementary Fig. 30a, d**), indicating the complete dissolution and restructuring of the original MnO_2 framework. Within the discharged cathode, Bi_2O_3 were still detected (**Supplementary Fig. 30b, c**), and the (002) lattice plane of β-Bi_2O_3 was clearly observable; the Bi_2O_3 particles had aggregated and increased in size, suggesting they likely detached from their original positions and recrystallized with the MnO_2 dissolution.

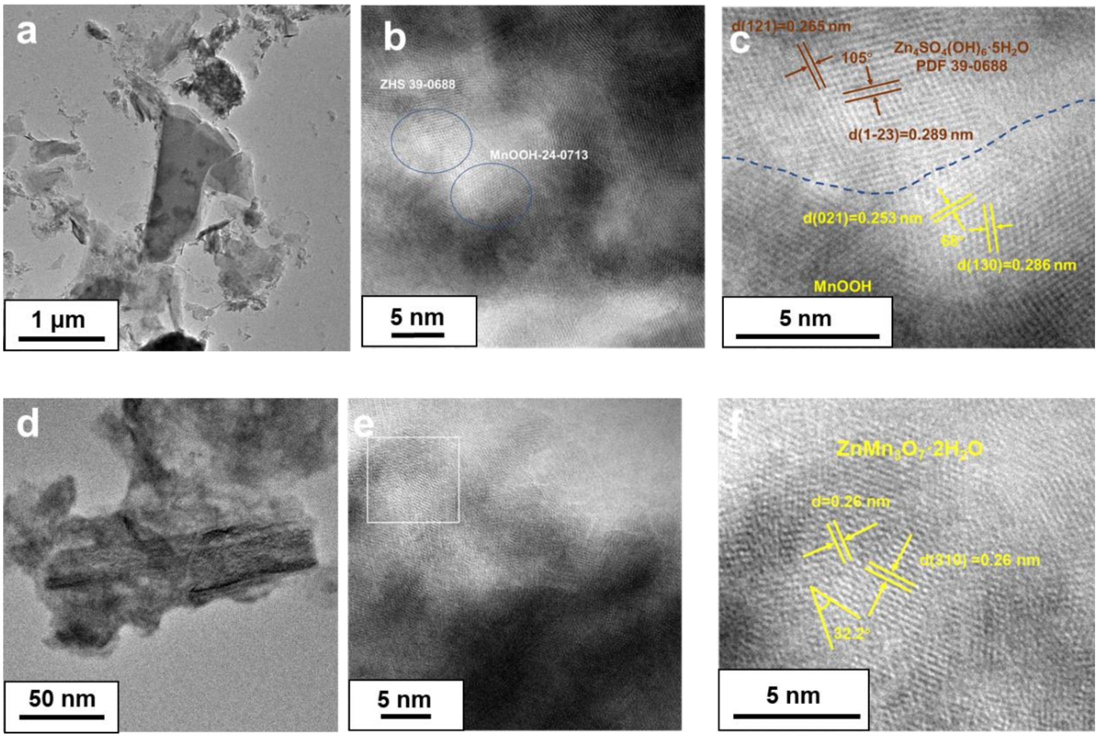

Supplementary Fig. 31: HRTEM images of MnO_2@BiO cathode at 10th cycle. **a**, **b**, **c** 10th discharged state and **d**, **e**, **f** 10th charged state. And the identification of phases in the fully discharged and charged cathodes.

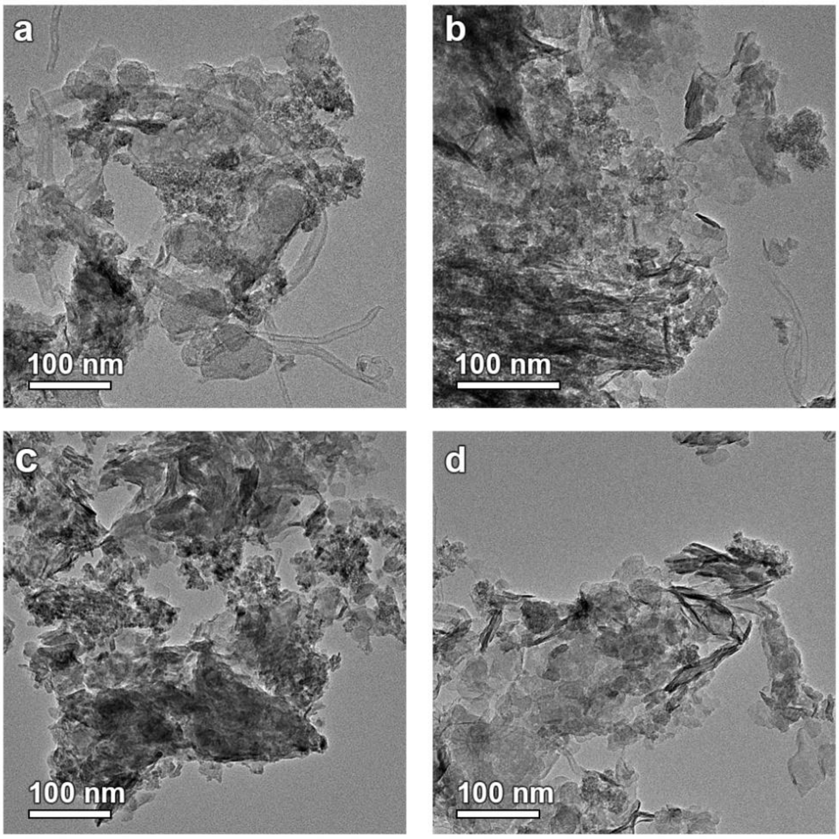

Supplementary Fig. 32: HRTEM images of cathodes at 100th cycle. **a** MnO_2@BiO at 100th discharged state, **b** MnO_2@BiO at 100th charged state, **c** MnO_2 at 100th discharged state, **d** MnO_2 at 100th charged state.

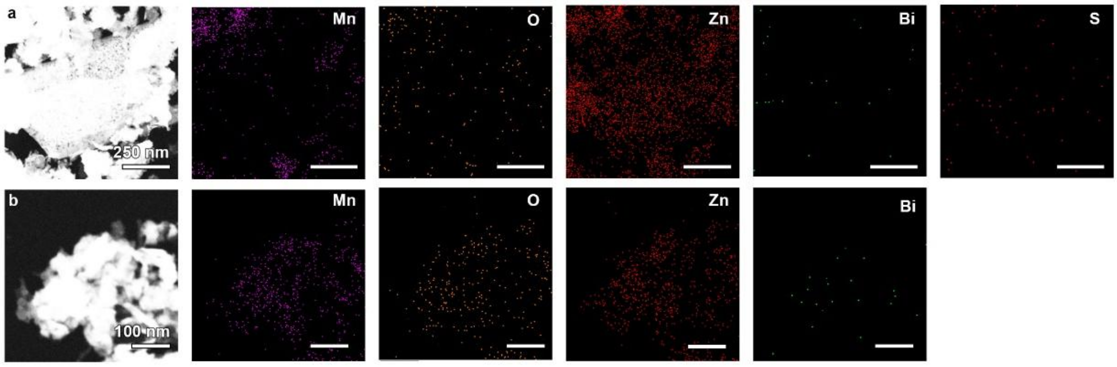

Supplementary Fig. 33: HAADF-STEM images and corresponding EDS maps of MnO_2@BiO at 100th cycle, including **a** 100th discharge, **b** 100th charge.

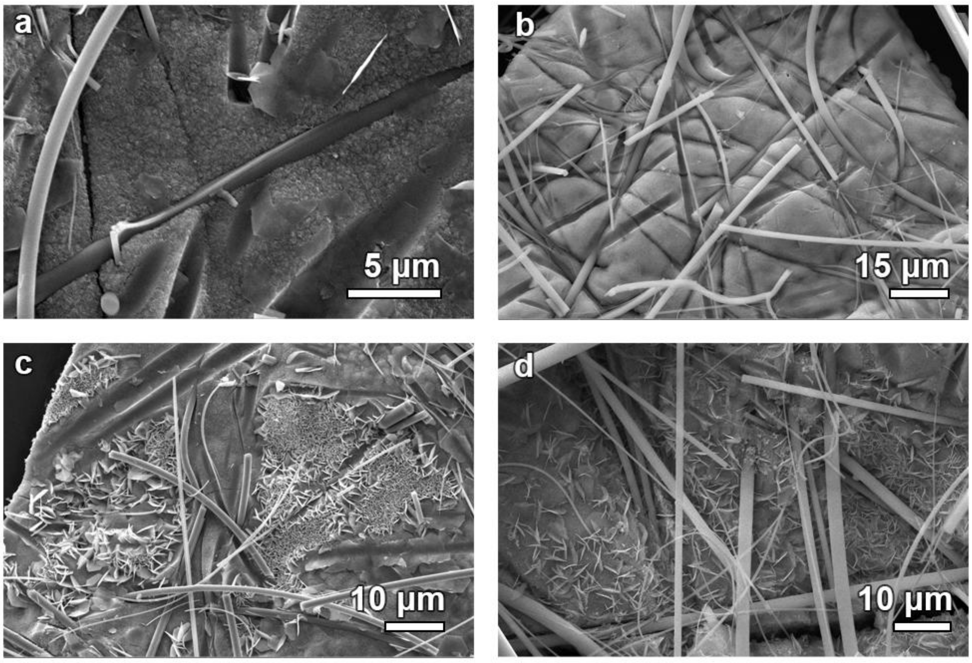

Supplementary Fig. 34: SEM images of cathodes in the extended cycles. **a** MnO_2 at 10th charged state, **b** MnO_2 at 100th charged state **c** MnO_2@BiO at 10th charged state, **d** MnO_2@BiO at 100th charged state.

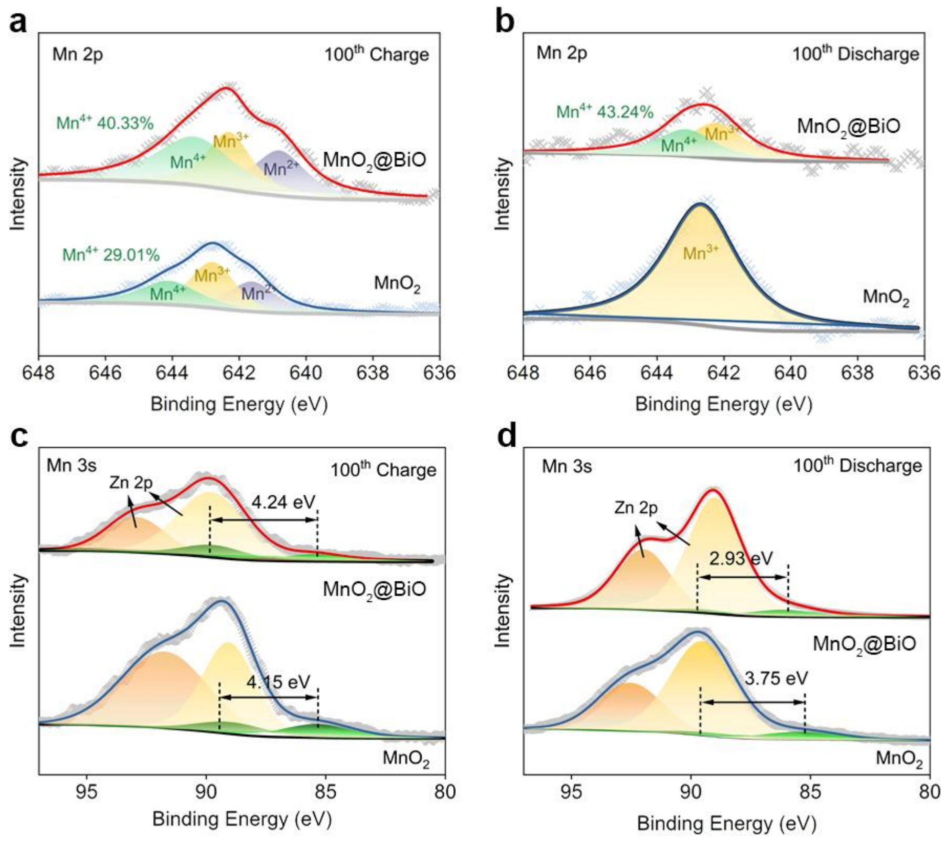

Supplementary Fig. 35: High-resolution Mn 2p and Mn 3s XPS spectra of the cathodes. **a** 100th charged, **b** 100th discharged states of Mn 2p XPS spectra, **c** 100th charged, **d** 100th discharged states of Mn 3s XPS spectra.

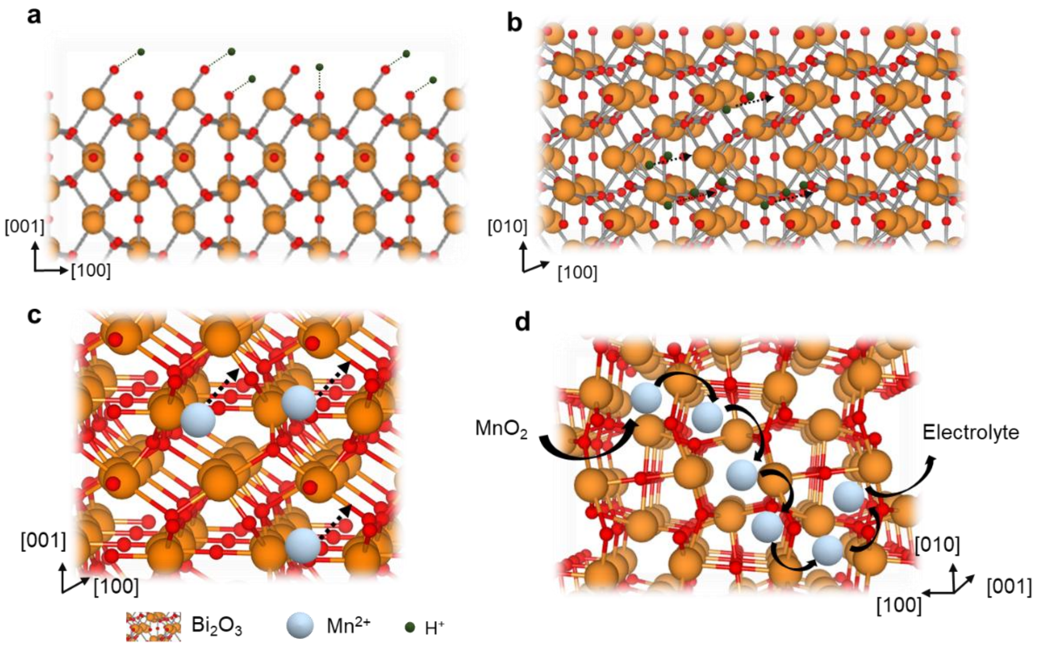

Supplementary Fig. 36: Schematic diagrams of H^+ diffusion in Bi_2O_3: **a** b-axis and **b** a-axis views of Bi_2O_3. Schematic diagrams of Mn^{2+} diffusion in Bi_2O_3: **c** along the a-axis and **d** the cross-section along the a-axis.

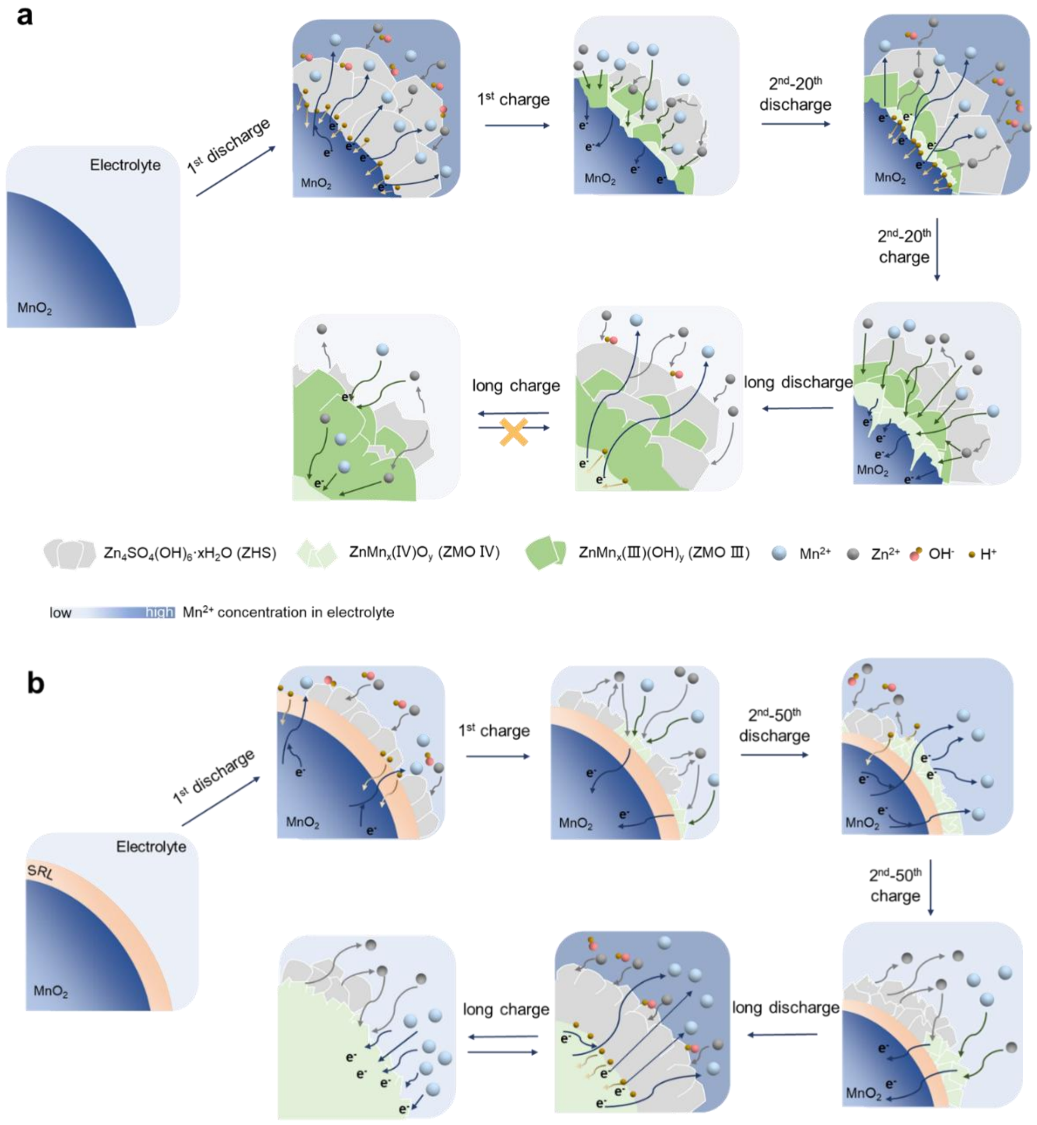

Supplementary Fig. 37: Schematic diagrams of discharge/charge processes from 1st cycle to long cycles of **a** MnO_2 and **b** MnO_2@BiO.

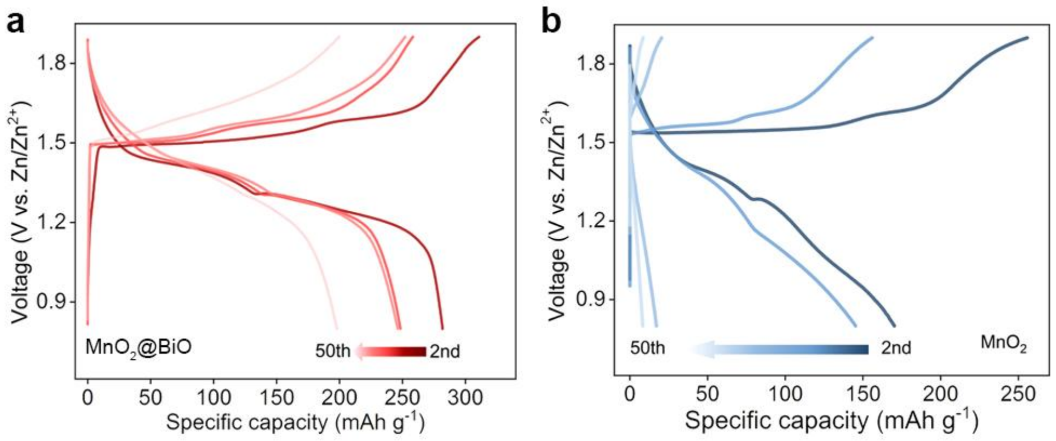

Supplementary Fig. 38: The charging/discharging plateaus of **a** MnO_2 cathode and **b** MnO_2@BiO cathode in 2 M $ZnSO_4$/0.2 M $MnSO_4$ electrolyte at 0.2 C.

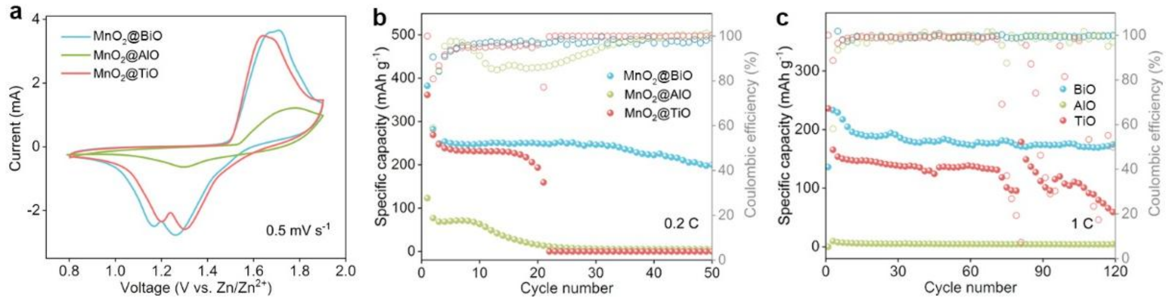

Supplementary Fig. 39: a CV profiles of BiO, AlO and TiO cathodes at 3rd cycle, with a scan rate of 0.5 mV s^{-1}. Cycling performance of BiO, AlO and TiO cathodes in 2 M $ZnSO_4$/0.2 M $MnSO_4$ electrolyte at the current density of **b** 0.2 C and **c** 1 C. The active material mass loading is around 9 mg cm^{-2}.

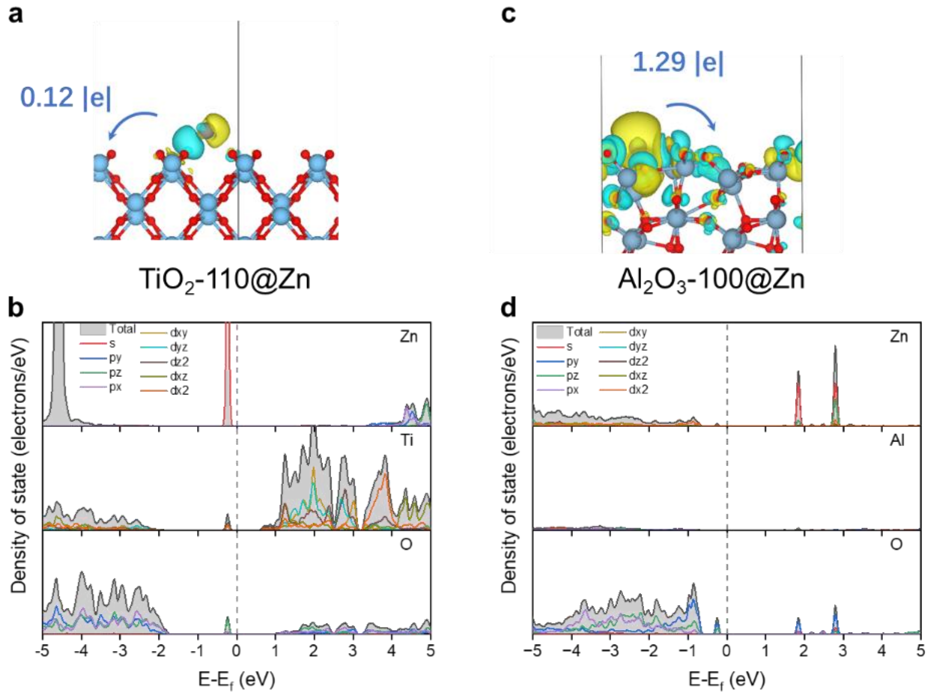

Supplementary Fig. 40: a Charge density difference of TiO_2@Zn, **b** DOS curve of TiO_2@Zn, **c** charge density difference of Al_2O_3@Zn, **d** DOS curve of Al_2O_3@Zn.

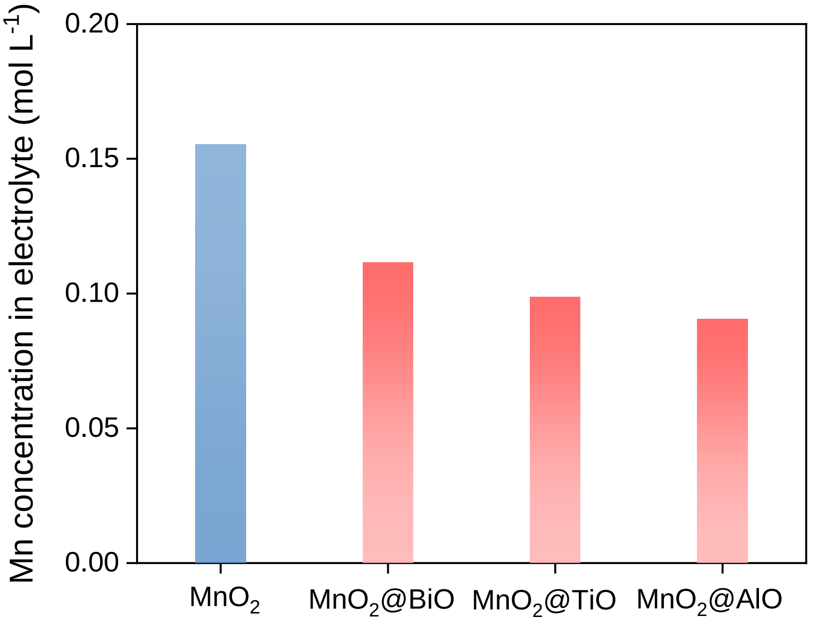

Supplementary Fig. 41: Mn ions concentration in electrolyte after 10th full discharged of MnO_2, MnO_2@BiO, MnO_2@TiO and MnO_2@AlO cathodes.

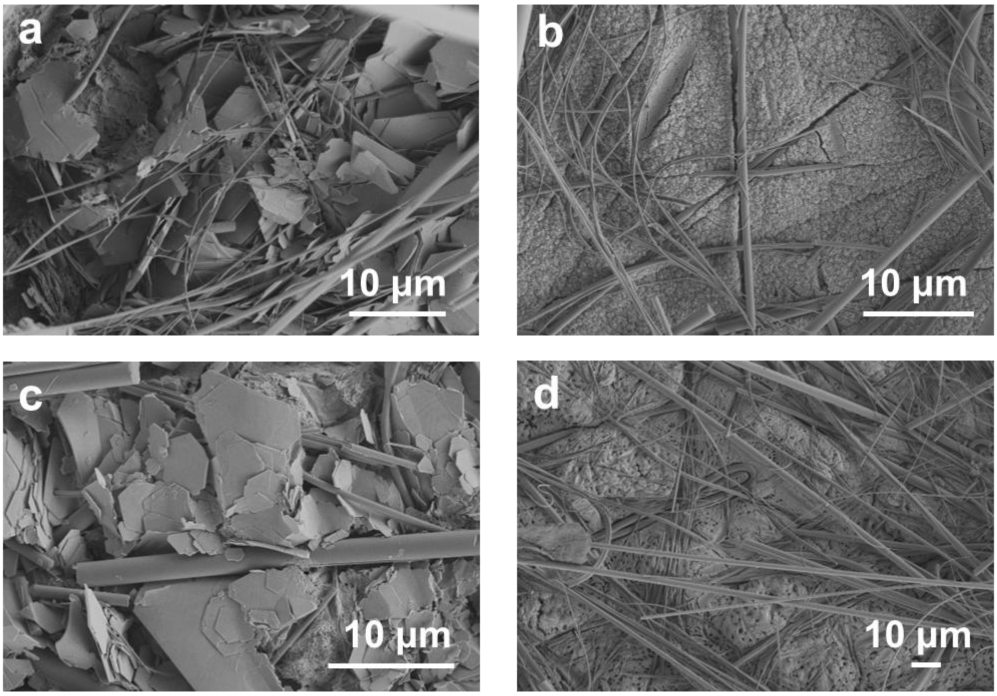

Supplementary Fig. 42: SEM images of MnO_2@AlO cathodes after **a** 10th discharged, **b** 10th charged, **c** 100th discharged and **d** 100th charged.

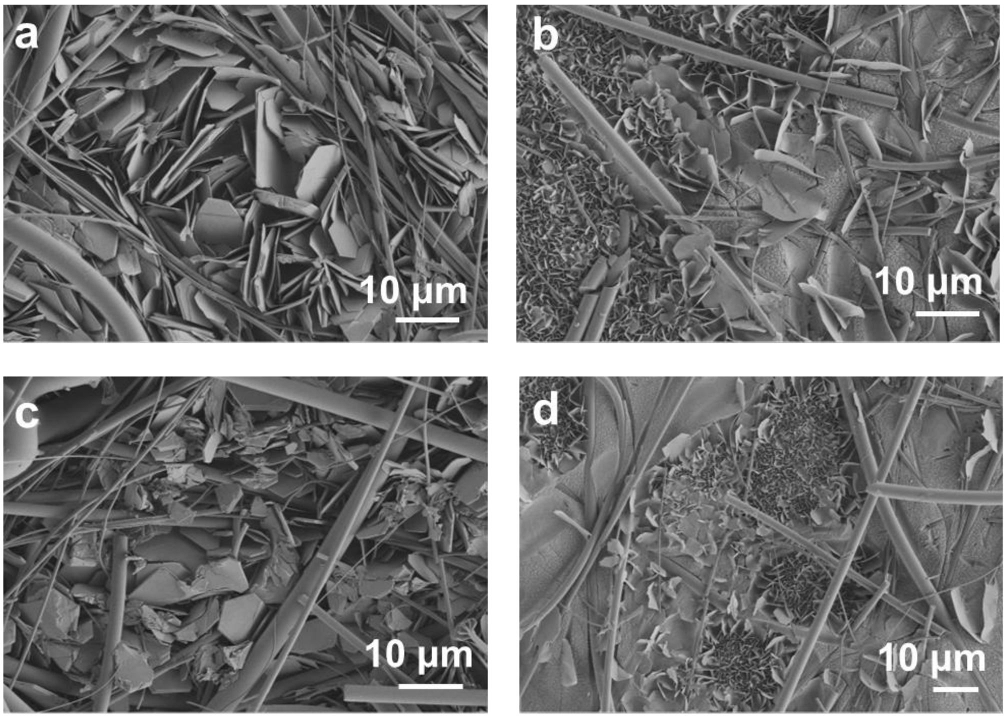

Supplementary Fig. 43: SEM images of MnO_2@TiO cathodes after **a** 10th discharged, **b** 10th charged, **c** 100th discharged and **d** 100th charged at 2 C rate.

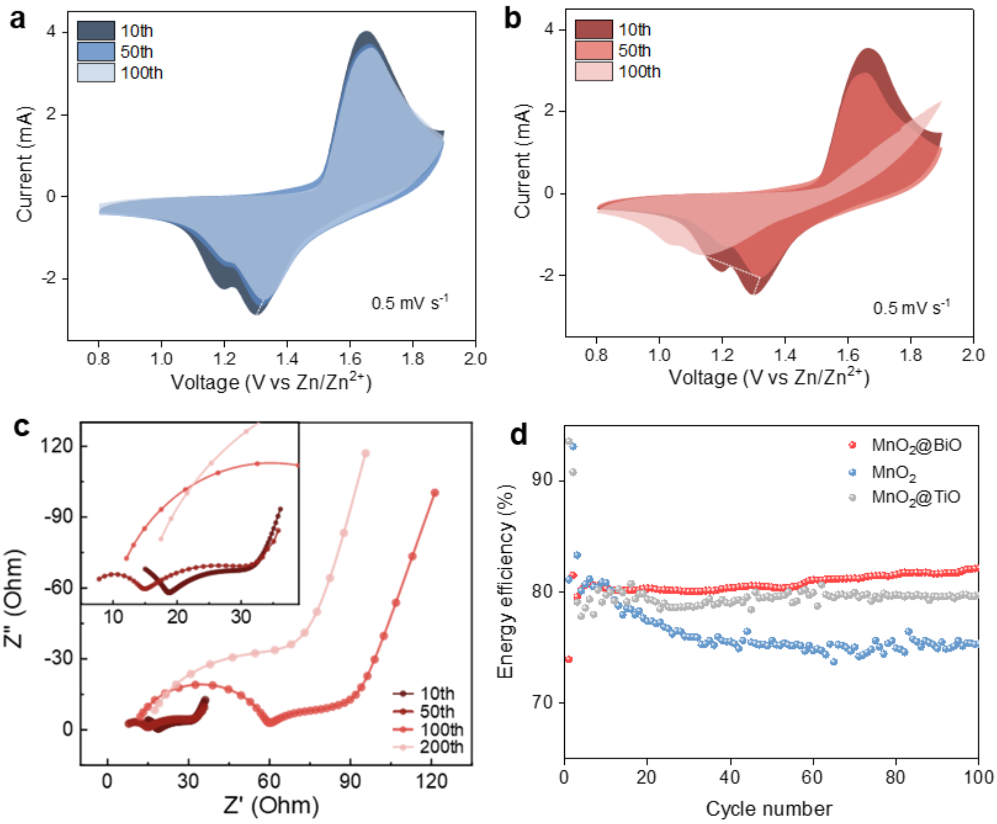

Supplementary Fig. 44: CV profiles of **a** MnO_2@BiO and **b** MnO_2@TiO cathodes at selected cycle number. **c** Nyquist plots of MnO_2@TiO cathodes over the frequency range of 100 mHz-1 MHz at selected cycles. **d** Energy efficiency of MnO_2@BiO, MnO_2@TiO and MnO_2 cathodes at 2 C rate.

Supplementary Note 1.

Fig. 1d reveals that Bi_2O_3 exhibits the highest Zn adsorption energy (4.48 eV) among the three materials, while TiO_2 shows the weakest adsorption. Notably, Al_2O_3 demonstrates adsorption energy comparable to Bi_2O_3, indicating its potential as a substrate for fostering small-sized product growth. **Supplementary Fig. 40** presents differential charge and density of states (DOS) analyses post-Zn adsorption. For Bi_2O_3, strong bonding interactions between Zn and adjacent Bi/O atoms are observed. In Al_2O_3, strong Zn-O interactions within the lattice are evident, whereas TiO_2 fails to achieve effective orbital coupling with Zn. The physical barrier function essential for SRL was evaluated across three materials, focusing on their ability to decelerate Mn^{2+} dissolution into the electrolyte and mitigate H^+ intercalation into MnO_2, thereby establishing an initial electrode/electrolyte interfacial microenvironment with optimal ion concentration. Batteries assembled with cathodes coated by the three materials underwent Inductively Coupled Plasma (ICP) analysis to quantify Mn-ion concentration in the electrolyte after full discharge (**Supplementary Fig. 41**). Results confirm that all three coating materials effectively function as ion barriers during MnO_2 electrode discharge, preventing sudden surges of Mn^{2+} concentration in the microenvironment. Post-cycling electrode morphology was examined by SEM images, displaying discharged/charged states of MnO_2@AlO and MnO_2@TiO electrodes after 10 and 100 cycles. For MnO_2@AlO electrodes (**Supplementary Fig. 42**), Al_2O_3's substantial Zn interaction enabled initial small-sized solid-phase products, but limited quantities. No distinct Mn-deposited flake products were observed on charged electrodes after extended cycling. In contrast, MnO_2@TiO electrodes (**Supplementary Fig. 43**) exhibited large-scale, high-density ZHS growth during discharge. Charged electrodes revealed extensive ZMO(III) deposits, demonstrating TiO_2's categorical lack of solid-phase product size modulation capability.

Based on 3rd CV profiles and cycling performance comparison (**Supplementary Fig. 39**), the initial capacity of MnO_2@TiO is comparable to that of MnO_2@BiO. And from the CV curve, the reversibility of the third cycle is even better than that of MnO_2, but its capacity rapidly declines upon further cycling (**Supplementary Fig. 44**),

similar to the behavior of bare MnO_2, indicating the degradation of cathode. This phenomenon arises because although TiO_2 provides physical barrier functionality to regulate interfacial concentration, its poor zincophilicity renders it incapable of facilitating nucleation of ZHS and ZMO, thereby failing to promote and sustain reversible solid-phase conversion. While Al_2O_3 demonstrates favorable zincophilicity, MnO_2@AlO consistently exhibits exceptionally low capacity, indicating complete suppression of electrochemical reactions. This primarily originates from insulating properties of Al_2O_3-characterized by a dielectric constant of merely 8-10 [1,2], and a wide bandgap of 8.7 eV [3]. In contrast, Bi_2O_3 and TiO_2 possess dielectric constants exceeding 150 [4,5] and 80 [6,7] respectively, enabling efficient electron transport to sustain electrochemical processes, whereas Al_2O_3 insulating layer impedes electron transfer. Additionally, **Supplementary Fig. 14** reveals significant lattice mismatch between Al_2O_3 and MnO_2, obstructing efficient ion channel formation and hindering Mn^{2+} dissolution for subsequent reactions. This impedes electrochemical reactions at the MnO_2 core and proves counterproductive to the goal of delaying MnO_2 electrode reconstruction. As summarized in **Table Note 1**, the multifaceted synergies in MnO_2@BiO collectively contribute to its exceptional long-cycle stability.

Table Note 1. Systematic comparison of Bi_2O_3, Al_2O_3 and TiO_2.

Layer materials	Physical barrier	Ions transfer	Electrons transfer	Products size regulation	Cyclic stability
Bi_2O_3	✓	✓	✓	✓	✓
Al_2O_3	✓	×	×	✓	×
TiO_2	✓	✓	×	×	×

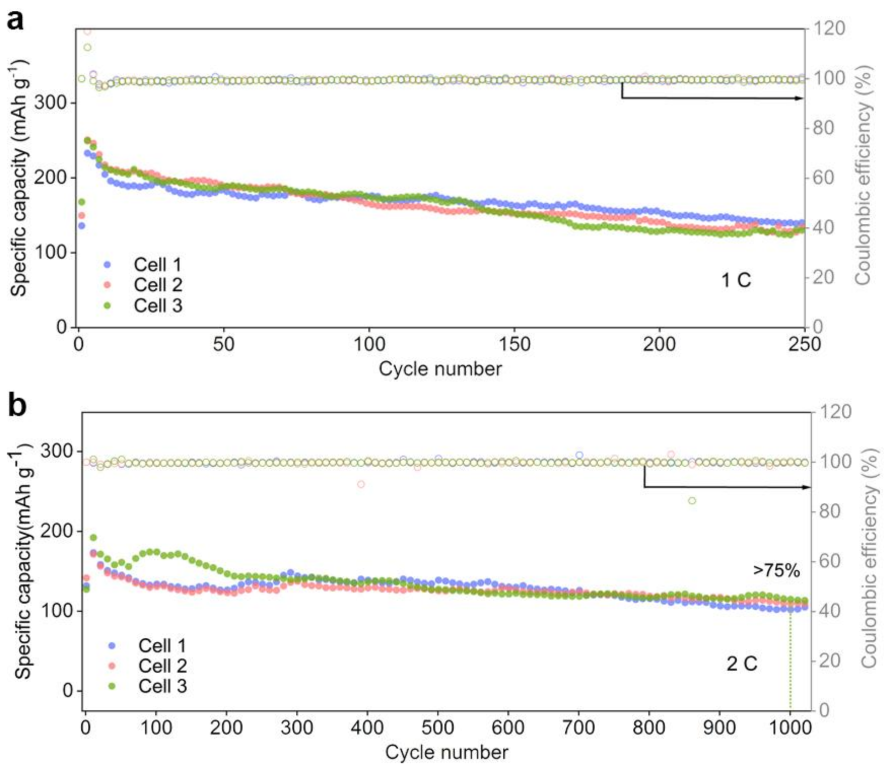

Supplementary Fig. 45: Cycling performance of MnO_2@BiO cathodes in 2 M $ZnSO_4$/0.2 M $MnSO_4$ electrolyte at **a** 1 C and **b** 2 C.

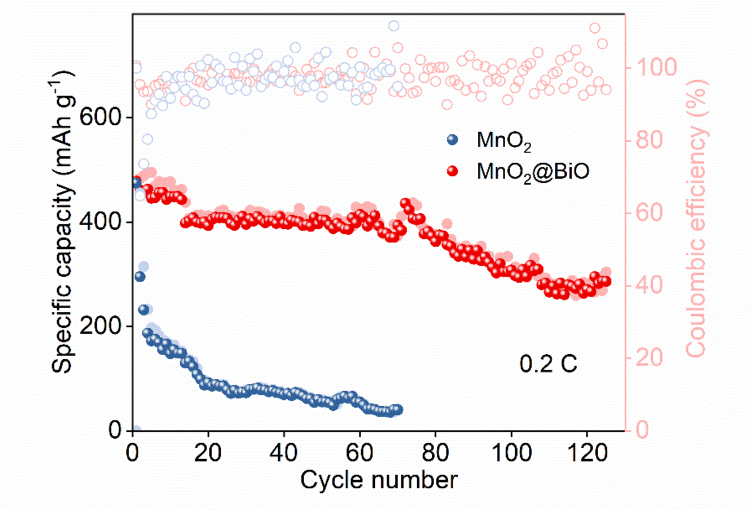

Supplementary Fig. 46: Cycling performance of MnO_2 and MnO_2@BiO cathodes in 2 M $ZnSO_4$/0.2 M $MnSO_4$ electrolyte at 0.2 C. The active material mass loading >9 mg cm^{-2}.

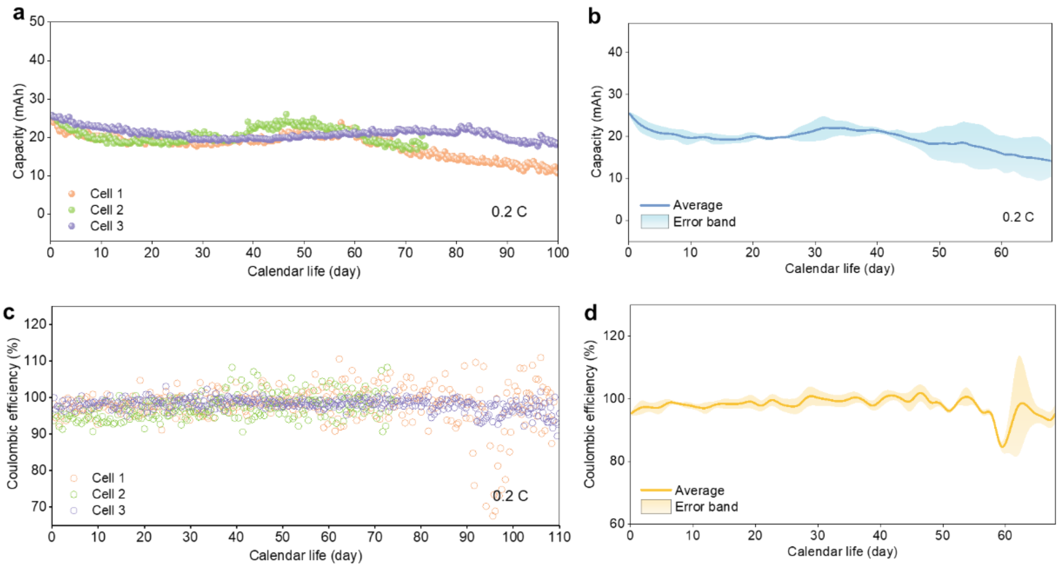

Supplementary Fig. 47: a Cycling performance of MnO_2@BiO cathodes in 2 M $ZnSO_4$/0.2 M $MnSO_4$ electrolyte at 0.2 C in iron-plate batteries and **b** corresponding error bands. **c** the Coulombic efficiencies and **d** corresponding error bands.

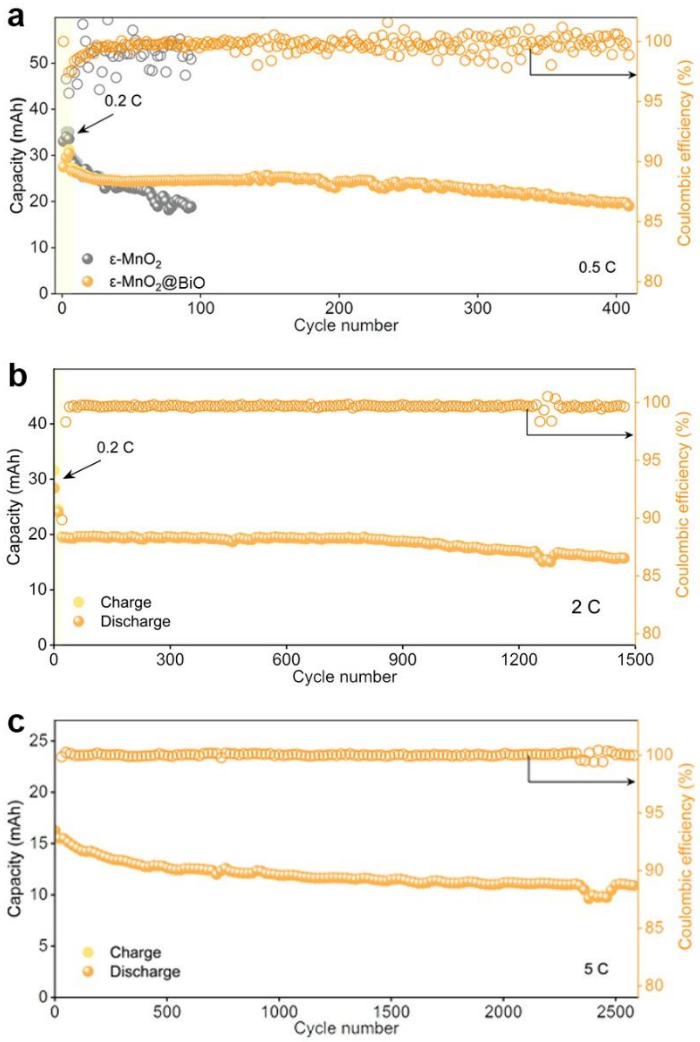

Supplementary Fig. 48: Cycling performance of high mass loading BiO-coated ε-MnO_2 (ε-MnO_2@BiO) cathodes in large batteries. Cycling performance of ε-MnO_2 and ε-MnO_2@BiO cathodes in 2 M $ZnSO_4$/0.2 M $MnSO_4$ electrolyte at the current densities of **a** 0.5 C, **b** 2 C and **c** 5 C. The active material mass loading >20 mg cm^{-2}.

Supplementary Tables

Supplementary Table 1. Comparison of cost budget and reaction conditions of different methods

Methode	Materials expenses		Add quantity	Treatment
Electrolyte engineering	Magnesium aluminosilicate-based colloidal electrolyte [8]	60.6 $/kg	40 wt%<br>0.4 g/mL	---
	SiO_2 and $FeSO_4$ mediator colloidal electrolyte [9]	SiO_2 30.3 $/kg<br>$FeSO_4$<br>215.8 $/kg	75 mg/mL<br>0.02 M	---
	ZnO gel-like electrolyte [10]	ZnO 26.8 $/kg	0.2 g/mL	---
Electrolyte additive (CEI)	Dioctyl Phthalate [11]	19.0 $/L	25% volume<br>0.15 mL coin<br>20 mL pouch	---
	Diethylenetriamine pentaacetate sodium salt [12]	58.7 $/kg	0.5 wt%	---
	Methacrylic acid [13]	20.7 $/L	2 M	Ultraviolet lamp for 2 h
Redox mediators	Iodide redox mediator [14]	KI 496.7 $/kg	0.1 M<br>2 mL electrolyte	---
	Bromine redox mediato [15]	$ZnBr_2$<br>190.6 $/kg<br>$Br_2$ 81.0 $/kg	0.05 M<br>0.2 M	---
	Fe^{2+} redox mediator [16]	$FeSO_4$ 215.8 $/kg	0.05 M	Purpose-made flow cell
Doping	Multi-ions doping (Cr^{3+}, Ni^{2+}) [17]	$Cr(CH_3COO)_3$<br>18.4 $/kg<br>$Ni(NO_3)_2{\cdot}6H_2O$<br>112.9 $/kg	0.01 M<br>0.01 M	In-situ electro-deposition
	Ca^{2+} pillars preintercalation [18]	$CaCl_2$<br>142.5 $/kg	$Ca_{0.46}Mn_{0.93\pm0.07}O_2$	Hydrothermal method
	Dual-anions doping (N, S) [19]	Thiourea<br>57.4 $/kg	---	Calcination in two zone tube furnaces
Heterostructure	MnS/MXene [20]	Ethylenediamine<br>42.7 $/L<br>$Mn(Ac)_2 \cdot 4H_2O$<br>128.9 $/kg<br>$Ti_3C_2T_x$ 27.4 $/g	---	Hydrothermal under Ar gas, dialyzed and freeze-dried

	BMO/MnO_2 [21]	$Bi(NO_3)_3{\cdot}5H_2O$ 71.1 $/kg Citric acid 32.8 $/kg	$Bi_{12.53}Mn_{0.47}O_{19.85}$/R-$MnO_2$	Hydrothermal method
Size revitalizing layer	Bi_2O_3 layer coated on MnO_2	$Bi(NO_3)_3{\cdot}5H_2O$ 71.1 $/kg	3 wt%	One-step calcination

Supplementary Table 2. Adsorption energy of Zn by different oxides.

**Oxide material**	**Space group number**	**Lattice plane**	**Eds (eV)**
MnO_2	136	110	1.42
TiO_2	141	110	0.32
Al_2O_3	194	100	-4.24
Co_3O_4	227	220	0.43
ZnO	186	100	0.4
Cr_2O_3	167	104	0.54
VO_2	136	001	0.89
V_2O_3	167	104	0.87
ZrO_2	225	111	0.78
MgO	225	110	0.46
Sb_2O_3	227	400	0.63
In_2O_3	206	022	-1.38
SnO_2	136	101	-2.59
Bi_2O_3	114	100	-4.48

Supplementary Table 3. Impedance results of MnO_2, BiO, AlO and TiO cathodes in 2 M $ZnSO_4$/0.2 M $MnSO_4$ electrolyte.

MnO_2

	R_s (Ω)	R_i (Ω)	R_{ct} (Ω)
**10th**	7.32	/	16.7
**50th**	5.00	/	127
**100th**	6.01	/	129
**200th**	2.71	/	147

MnO_2@BiO

	R_s (Ω)	R_i (Ω)	R_{ct} (Ω)
**10th**	12.5	4.15	8.62
**50th**	6.67	6.94	16.1
**100th**	7.45	4.51	13.7
**200th**	8.39	4.86	11.9

MnO_2@AlO

	R_s (Ω)	R_i (Ω)	R_{ct} (Ω)
**10th**	6.46	27.5	103
**50th**	10.8	/	66.6
**100th**	11.1	/	73.1
**200th**	38.8	/	78.8

MnO_2@TiO

	R_s (Ω)	R_i (Ω)	R_{ct} (Ω)
**10th**	1.82	16.6	17.5
**50th**	5.76	8.45	22.4
**100th**	9.91	49.3	21.0
**200th**	14.5	/	67.9

Supplementary Table 4. Cycling performance of MnO_2@BiO electrodes in 2 M $ZnSO_4$/0.2 M $MnSO_4$ electrolyte at different current densities.

**Current density**	**Number of cells**	**Average specific capacity (mAh g^{-1})**	**Average Coulombic efficiency (%)**
**1 C** (within 200 cycles)	1	173.955	99.417
	2	173.204	99.487
	3	172.395	99.395
**2 C** (within 1000 cycles)	1	128.786	99.899
	2	126.196	99.788
	3	134.949	99.974

Supplementary Table 5. Electrochemical performances of recently reported manganese-based cathode materials for zinc-ion battery.

Electrode material	Electrolyte	Mass loading	Capacity	Current density	Capacity retention	Ref
Sn_xMnO_2/SnO_2	2 M $ZnSO_4$/0.1 M $MnSO_4$	0.8 mg cm^{-2}	0.27 mAh	0.30 A g^{-1}	100%/100 cycles	A [22]
d-MnO_2@CC	2 M $ZnSO_4$/0.1 M $MnSO_4$	1.1 mg cm^{-2}	0.30 mAh	0.25 A g^{-1}	100%/245 cycles	B [23]
K-V_2C@MnO_2	2 M $ZnSO_4$/0.25 M $MnSO_4$	1.2 mg cm^{-2}	0.61 mAh	0.30 A g^{-1}	100%/180 cycles	C [24]
α-MnO_2@g-C_3N_4	2 M $ZnSO_4$/0.1 M $MnSO_4$	1.7 mg cm^{-2}	0.55 mAh	0.50 A g^{-1}	100%/225 cycles	D [25]
K-δ-MnO_2	2 M $ZnSO_4$/0.1 M $MnSO_4$	2.0 mg cm^{-2}	0.63 mAh	0.30 A g^{-1}	100%/100 cycles	E [26]
MnO_2@N	2 M $ZnSO_4$/0.5 M $MnSO_4$	2.0 mg cm^{-2}	0.39 mAh	0.50 A g^{-1}	100%/500 cycles	F [27]
HP-Mn_2O_3-YSMSs	2 M $ZnSO_4$/0.1 M $MnSO_4$	2.0 mg cm^{-2}	0.45 mAh	0.50 A g^{-1}	100%/200 cycles	G [28]
ε-MnO_2	1 M $ZnSO_4$/0.2 M $MnSO_4$	3.0 mg cm^{-2}	0.66 mAh	0.75 A g^{-1}	90.0%/500 cycles	H [29]
β-MnO_2	2 M $ZnSO_4$/0.1 M $MnSO_4$	4.0 mg cm^{-2}	0.79 mAh	0.50 A g^{-1}	45.0%/700 cycles	I [30]
D-β-MnO_2	3 M $ZnSO_4$/0.1 M $MnSO_4$	4.0 mg cm^{-2}	1.10 mAh	0.50 A g^{-1}	100%/300 cycles	J [31]
α-MnO_2	2 M $ZnSO_4$/0.2 M $MnSO_4$	4.0 mg cm^{-2}	0.90 mAh	0.50 A g^{-1}	52.6%/100 cycles	K [32]
MnO_2/rGO	2 M $ZnSO_4$/0.1 M $MnSO_4$	5.0 mg cm^{-2}	1.41 mAh	0.40 A g^{-1}	82.0%/100 cycles	L [33]
MnO_2/C	2 M $ZnSO_4$/0.5 M $MnSO_4$	5.0 mg cm^{-2}	1.64 mAh	0.60 A g^{-1}	100%/250 cycles	M [34]
S-MnO_2	2 M $ZnSO_4$/0.1 M $MnSO_4$	5.0 mg cm^{-2}	1.81 mAh	0.20 A g^{-1}	100%/100 cycles	N [35]
NMO/VTCNTs	2 M $ZnSO_4$/0.2 M $MnSO_4$	5.0 mg cm^{-2}	1.85 mAh	0.20 A g^{-1}	100%/120 cycles	O [36]
C@PODA/MnO_2	2 M $ZnSO_4$/0.1 M $MnSO_4$	6.8 mg cm^{-2}	1.47 mAh	0.50 A g^{-1}	100%/600 cycles	P [37]
$LiMn_2O_4$	1 M $ZnSO_4$/2 M Li_2SO_4	7.2 mg cm^{-2}	0.61 mAh	0.15 A g^{-1}	62.0%/500 cycles	Q [38]
NCMO	2 M $ZnSO_4$/0.2 M $MnSO_4$	7.5 mg cm^{-2}	1.53 mAh	0.75 A g^{-1}	100%/100 cycles	R [39]
3D $Ti_3C_2T_x$@MnO_2	1 M $ZnSO_4$	8.0 mg cm^{-2}	2.35 mAh	0.50 A g^{-1}	71.1%/600 cycles	S [40]

MnO_2/CNT	“soggy-sand”(2 M $ZnSO_4$/0.1 M $MnSO_4$/Al_2O_3/SiO_2)	10 mg cm^{-2}	2.71 mAh	0.50 A g^{-1}	100%/300 cycles	T [41]
S-MnOOH	2 M $ZnSO_4$/0.1 M $MnSO_4$	10 mg cm^{-2}	1.22 mAh	0.50 A g^{-1}	100%/500 cycles	U [42]
MnO_2@MXene	0.5 M $Zn(OTf)_2$/TEP	10.5 mg cm^{-2}	1.93 mAh	0.10 A g^{-1}	100%/100 cycles	V [43]
This work	2 M $ZnSO_4$/0.2 M $MnSO_4$	9.0 mg cm^{-2}	2.54 mAh	0.25 A g^{-1} (1 C)	80.1%/200 cycles	*
			1.96 mAh	0.50 A g^{-1} (2 C)	85.0%/1000 cycles	

Supplementary Table 6 Electrochemical performances of recently reported single-layer cathode pouch-type Zn-ion batteries.

Battery systems	Active material mass	Mass loading	Current density	Cell capacity	Energy density	Calendar life	Ref
P-Zn//VO_2	5.84 mg	0.649 mg cm^{-2}	1 A g^{-1}	1.17 mAh	75 Wh kg^{-1}	5.5 days/330 cycles	A [44]
Zn//PANI	16.5 mg	1.03 mg cm^{-2}	0.05 A g^{-1}	2.14 mAh	/	8.5 days/50 cycles	B [45]
Zn//MnO_2	18.0 mg	4.00 mg cm^{-2}	0.25 A g^{-1}	3.60 mAh	48.6 Wh kg^{-1}	17 days/200 cycles	C [46]
Zn//V_2O_5	21.0 mg	2.10 mg cm^{-2}	1 A g^{-1}	5.90 mAh	/	5.8 days/100 cycles	D [47]
Zn//NVO	52.0 mg	2.60 mg cm^{-2}	0.5 A g^{-1}	6.32 mAh	/	4.2 days/100 cycles	E [48]
Zn@C//V_2O_5	25.5 mg	1.70 mg cm^{-2}	1 A g^{-1}	7.65 mAh	354 Wh kg^{-1}	10 days/600 cycles	F [49]
Zn//MnO_2@Graphite	100 mg	2.00 mg cm^{-2}	2 A g^{-1}	8.50 mAh	/	4.2 days/50 cycles	G [50]
Zn//$NH_4V_4O_{10}$	52.5 mg	2.10 mg cm^{-2}	1 A g^{-1}	10.5 mAh	/	10 days/600 cycles	H [51]
SAM-Zn//$NH_4V_4O_{10}$	48.0 mg	4.00 mg cm^{-2}	2 A g^{-1}	10.1 mAh	/	8.3 days/200 cycles	I [52]
PCu@Zn//LMO	104 mg	10.5 mg cm^{-2}	0.15 A g^{-1}	10.0 mAh	/	25 days/300 cycles	J [53]
NGO@Zn//LMO	80.6 mg	10.5 mg cm^{-2}	0.15 A g^{-1}	10.0 mAh	164 Wh kg^{-1}	15 days/180 cycles	K [54]
ZAP@Zn@Cu// V_2O_5	60.0 mg	4.00 mg cm^{-2}	0.5 A g^{-1}	12.0 mAh	155.8 Wh kg^{-1}	6.7 days/200 cycles	L [55]
Zn//$NH_4V_4O_{10}$	45.0 mg	4.00 mg cm^{-2}	1 A g^{-1}	12.2 mAh	/	17 days/1000 cycles	M [56]
Zn@MCFs//α-MnO_2	57.6 mg	9.60 mg cm^{-2}	1 A g^{-1}	11.5 mAh	154.3 Wh kg^{-1}	5.2 day/250 cycles	N [57]
Zn//LMO	100 mg	11.0 mg cm^{-2}	0.15 A g^{-1}	12.5 mAh	/	33 days/400 cycles	O [58]

Zn//V ö-MnO_2@PEDOT	57.6 mg	2.40 mg cm^{-2}	0.2 A g^{-1}	17.8 mAh	/	10 days/120 cycles	P [59]
Zn//ZnVOH	78.8 mg	7.00 mg cm^{-2}	1 mA cm^{-2}	20.7 mAh	/	28 days/200 cycles	Q [60]
3DGT@Zn//V_2O_5	42.8 mg	11.4 mg cm^{-2}	4 mA cm^{-2}	20.5 mAh	286.8 Wh kg^{-1}	17 days/150 cycles	R [61]
This work	121 mg	16 mg cm^{-2}	0.05 A g^{-1}	23.1 mAh	284.9 Wh kg^{-1}	110 days/350 cycles	*

Supplementary References

1. Panasci SE, Schilirò E, Greco G et al. Strain, Doping, and Electronic Transport of Large Area Monolayer MoS_2 Exfoliated on Gold and Transferred to an Insulating Substrate. *ACS Appl Mater Interfaces* 2021; **13**: 31248-31259.

2. Dong H, Zhang C, Nie W et al. Interfacial Chemistry Triggers Ultrafast Radiative Recombination in Metal Halide Perovskites. *Angew Chem Int Ed* 2022; **61**: e202115875.

3. Chaiseeda K, Nishimura S, Ebitani K. Gold Nanoparticles Supported on Alumina as a Catalyst for Surface Plasmon-Enhanced Selective Reductions of Nitrobenzene. *ACS Omega* 2017; **2**: 7066-7070.

4. Liu Z, Qin M, Fu B et al. Effective Proton Conduction in Quasi-Solid Zinc-Manganese Batteries via Constructing Highly Connected Transfer Pathways. *Angew Chem Int Ed* 2025; **64**: e202417049.

5. Wu Z, Liao T, Wang S et al. Conversion of Catalytically Inert 2D Bismuth Oxide Nanosheets for Effective Electrochemical Hydrogen Evolution Reaction Catalysis via Oxygen Vacancy Concentration Modulation. *Nano-Micro Lett* 2022; **14**: 90.

6. Pyeon JJ, Cho CJ, Jeong DS et al. A Ru-Pt alloy electrode to suppress leakage currents of dynamic random-access memory capacitors. *Nanotechnology* 2018; **29**: 455202.

7. Bae K-L, Kim J, Lim CK et al. Colloidal zinc oxide-copper(I) oxide nanocatalysts for selective aqueous photocatalytic carbon dioxide conversion into methane. *Nat Commun* 2017; **8**: 1156.

8. Zhou C, Xu Z, Nan Q et al. Spatial Confinement Effect of Mineral-Based Colloid Electrolyte Enables Stable Interface Reaction for Aqueous Zinc-Manganese Batteries. *Adv Energy Mater* 2025; **15**: 2405387.

9. Chen H, Ruan P, Zhang H et al. Achieving Highly Reversible Mn^{2+}/MnO_2 Conversion Reaction in Electrolytic Zn-MnO_2 Batteries via Electrochemical-Chemical Process Regulation. *Angew Chem Int Ed* 2025; **64**: e202423999.

10. Wu J, Tang Y, Xu H et al. ZnO Additive Boosts Charging Speed and Cycling Stability of Electrolytic Zn-Mn Batteries. *Nano-Micro Lett* 2024; **16**: 74.

11. Lai G, Zhao Z, Zhang H et al. In-situ positive electrode-electrolyte interphase construction enables stable Ah-level Zn-MnO_2 batteries. *Nat Commun* 2025; **16**: 2194.

12. Huang Y, Yan H, Liu W et al. Transforming Zinc-Ion Batteries with DTPA-Na: A Synergistic SEI and CEI Engineering Approach for Exceptional Cycling Stability and Self-Discharge Inhibition. *Angew Chem Int Ed* 2024; **63**: e202409642.

13. Li X, Qi K, Qin Z et al. Ion-Anchored Strategy for MnO_2/Mn^{2+} Chemistry without “Dead Mn” and Corrosion. *ACS Nano* 2024; **18**: 27016-27025.

14. Lei J, Yao Y, Wang Z et al. Towards high-areal-capacity aqueous zinc-manganese batteries: promoting MnO_2 dissolution by redox mediators. *Energy Environ Sci* 2021; **14**: 4418-4426.

15. Zheng X, Wang Y, Xu Y et al. Boosting Electrolytic MnO_2-Zn Batteries by a Bromine Mediator. *Nano Lett* 2021; **21**: 8863-8871.

16. Wang Q, Zhou W, Zhang Y et al. Rescue of dead MnO2 for stable electrolytic Zn-Mn redox-flow battery: a metric of mediated and catalytic kinetics. *Natl Sci Rev* 2024; **11**.

17. Zhao Y, Xia X, Li Q et al. Activating the redox chemistry of MnO_2/Mn^{2+} in aqueous Zn batteries based on multi-ions doping regulation. *Energy Storage Mater* 2024; **67**: 103268.

18. Wang T, Jin J, Zhao X et al. Unraveling the Anionic Redox Chemistry in Aqueous Zinc-MnO_2 Batteries. *Angew Chem Int Ed* 2024; **63**: e202412057.

19. Liang J, Zhao Y, Ren L et al. Dual Anions Doping Enhanced Conductivity and Stability of Layered δ-MnO_2 Cathode for Aqueous Zinc-Ion Battery. *Adv Funct Mater* 2025; **35**: 2501135.

20. Zhu Y, Zhao R, Xu Y et al. Anion Vacancies Coupling with Heterostructures Enable Advanced Aerogel Cathode for Ultrafast Aqueous Zinc-Ion Storage. *Adv Mater* 2025; **37**: 2419582.

21. Zhao X, Zhang F, Li H et al. Dynamic heterostructure design of MnO_2 for high-performance aqueous zinc-ion batteries. *Energy Environ Sci* 2024; **17**: 3629-3640.

22. Wang S, Ma W, Sang Z et al. Dual-modification of manganese oxide by heterostructure and cation pre-intercalation for high-rate and stable zinc-ion storage. *J Energy Chem* 2022; **67**: 82-91.

23. Shang P, Liu Y, Mei Y et al. Defective MnO_2 nanosheets based free-standing and high mass loading electrodes for high energy density aqueous zinc ion batteries. *Mater Chem Front* 2021; **5**: 8002-8009.

24. Zhu X, Cao Z, Wang W et al. Superior-Performance Aqueous Zinc-Ion Batteries Based on the In Situ Growth of MnO_2 Nanosheets on V_2CT_X MXene. *ACS Nano* 2021; **15**: 2971-2983.

25. Xie J, Liu G, Wang K et al. g-C_3N_4-coated MnO_2 hollow nanorod cathode for stable aqueous Zn-ion batteries. *Front Chem Sci Eng* 2023; **17**: 217-225.

26. Xie Q, Cheng G, Xue T et al. Alkali ions pre-intercalation of δ-MnO_2 nanosheets for high-capacity and stable Zn-ion battery. *Mater Today Energy* 2022; **24**: 100934.

27. Zhang Y, Liu Y, Liu Z et al. MnO_2 cathode materials with the improved stability via nitrogen doping for aqueous zinc-ion batteries. *J Energy Chem* 2022; **64**: 23-32.

28. Luo H, Wang B, Jian J et al. Stress-release design for high-capacity and long-time

lifespan aqueous zinc-ion batteries. *Mater Today Energy* 2021; **21**: 100799.

29. Huang L, Luo X, Chen C et al. A high specific capacity aqueous zinc-manganese battery with a ε-MnO_2 cathode. *Ionics* 2021; **27**: 3933-3941.

30. Liao Y, Chen H-C, Yang C et al. Unveiling performance evolution mechanisms of MnO_2 polymorphs for durable aqueous zinc-ion batteries. *Energy Storage Mater* 2022; **44**: 508-516.

31. Han M, Huang J, Liang S et al. Oxygen Defects in β-MnO_2 Enabling High-Performance Rechargeable Aqueous Zinc/Manganese Dioxide Battery. *iScience* 2020; **23**: 100797.

32. Gao X, Wu H, Li W et al. H^+-Insertion Boosted α-MnO_2 for an Aqueous Zn-Ion Battery. *Small* 2020; **16**: 1905842.

33. Wang J, Wang J-G, Liu H et al. A Highly Flexible and Lightweight MnO2/Graphene Membrane for Superior Zinc-Ion Batteries. *Adv Funct Mater* 2021; **31**: 2007397.

34. Li G, Huang Z, Chen J et al. Rechargeable Zn-ion batteries with high power and energy densities: a two-electron reaction pathway in birnessite MnO_2 cathode materials. *J Mater Chem A* 2020; **8**: 1975-1985.

35. Zhao Y, Zhang P, Liang J et al. Uncovering sulfur doping effect in MnO_2 nanosheets as an efficient cathode for aqueous zinc ion battery. *Energy Storage Mater* 2022; **47**: 424-433.

36. Gao X, Zhang C, Dai Y et al. Three-Dimensional Manganese Oxide@Carbon Networks as Free-Standing, High-Loading Cathodes for High-Performance Zinc-Ion Batteries. *Small Struct* 2023; **4**: 2200316.

37. Zhao Y, Zhou R, Song Z et al. Interfacial Designing of MnO_2 Half-Wrapped by Aromatic Polymers for High-Performance Aqueous Zinc-Ion Batteries. *Angew Chem Int Ed* 2022; **61**: e202212231.

38. Zhang Y, Zhang Y, Deng J et al. In Situ Electrochemically-Bonded Self-Adapting Polymeric Interface for Durable Aqueous Zinc Ion Batteries. *Adv Funct Mater* 2024; **34**: 2310995.

39. Gao X, Shen C, Dong H et al. Co-intercalation strategy for simultaneously boosting two-electron conversion and bulk stabilization of Mn-based cathodes in aqueous zinc-ion batteries. *Energy Environ Sci* 2024; **17**: 2287-2297.

40. Shi M, Wang B, Chen C et al. 3D high-density MXene@MnO_2 microflowers for advanced aqueous zinc-ion batteries. *J Mater Chem A* 2020; **8**: 24635-24644.

41. Deng R, Chen J, Chu F et al. "Soggy-Sand" Chemistry for High-Voltage Aqueous Zinc-Ion Batteries. *Adv Mater* 2024; **36**: 2311153.

42. Chen L, Dong Y, Han M et al. Adjusting Zn^{2+} diffusion kinetics and storage capability of MnOOH nanofibers for high-performance cathode of aqueous zinc-ion batteries.

Chem Eng J 2023; **473**: 145046.

43. Wu L, Mei Y, Liu Y et al. Interfacial synthesis of strongly-coupled δ-MnO_2/MXene heteronanosheets for stable zinc ion batteries with Zn^{2+}-exclusive storage mechanism. *Chem Eng J* 2023; **459**: 141662.

44. Zhang Q, Liang J, Li M et al. Stable zinc anode with ionic conductive interface layer for high performance aqueous zinc-ion batteries. *Chem Eng J* 2023; **474**: 145981.

45. Geng L, Meng J, Wang X et al. Eutectic Electrolyte with Unique Solvation Structure for High-Performance Zinc-Ion Batteries. *Angew Chem Int Ed* 2022; **61**: e202206717.

46. Shi S, Zhou D, Jiang Y et al. Lightweight Zn-Philic 3D-Cu Scaffold for Customizable Zinc Ion Batteries. *Adv Funct Mater* 2024; **34**: 2312664.

47. Bai X, Nan Y, Yang K et al. Zn Ionophores to Suppress Hydrogen Evolution and Promote Uniform Zn Deposition in Aqueous Zn Batteries. *Adv Funct Mater* 2023; **33**: 2307595.

48. Woottapanit P, Yang C, Cao J et al. Inhibition of Zinc Dendrite Growth by WC-Cellulose Separators for High-Performance Zinc-Ion Batteries. *ACS Appl Energy Mater* 2023; **6**: 10578-10584.

49. Zhang F, Liao T, Liu C et al. Biomineralization-inspired dendrite-free Zn-electrode for long-term stable aqueous Zn-ion battery. *Nano Energy* 2022; **103**: 107830.

50. Cao J, Zhang D, Gu C et al. Modulating Zn deposition via ceramic-cellulose separator with interfacial polarization effect for durable zinc anode. *Nano Energy* 2021; **89**: 106322.

51. Zhu X, Wei T, Zhang X et al. Tailoring the Solvation Environment Enables Dendrite-Free Zn Anodes for Stable Zinc-Ion Batteries. *ACS Sustain Chem Eng* 2023; **11**: 16975-16983.

52. Li D, Tang Y, Liang S et al. Self-assembled multilayers direct a buffer interphase for long-life aqueous zinc-ion batteries. *Energy Environ Sci* 2023; **16**: 3381-3390.

53. Zhou J, Wu F, Mei Y et al. Establishing Thermal Infusion Method for Stable Zinc Metal Anodes in Aqueous Zinc-Ion Batteries. *Adv Mater* 2022; **34**: 2200782.

54. Zhou J, Xie M, Wu F et al. Ultrathin Surface Coating of Nitrogen-Doped Graphene Enables Stable Zinc Anodes for Aqueous Zinc-Ion Batteries. *Adv Mater* 2021; **33**: 2101649.

55. Yang N, Gao Y, Bu F et al. Backside Coating for Stable Zn Anode with High Utilization Rate. *Adv Mater* 2024; **36**: 2312934.

56. Yan T, Tao M, Liang J et al. Refining the inner Helmholtz plane adsorption for achieving a stable solid-electrolyte interphase in reversible aqueous Zn-ion pouch cells. *Energy Storage Mater* 2024; **65**: 103190.

57. Ying H, Huang P, Zhang Z et al. Freestanding and Flexible Interfacial Layer Enables

Bottom-Up Zn Deposition Toward Dendrite-Free Aqueous Zn-Ion Batteries. *Nano-Micro Lett* 2022; **14**: 180.

58. Li G, Zhao Z, Zhang S et al. A biocompatible electrolyte enables highly reversible Zn anode for zinc ion battery. *Nat Commun* 2023; **14**: 6526.

59. Chen H, Guo J, Tan S et al. Dual-modification of oxygen vacancies and PEDOT coating on MnO_2 nanowires for high-performance zinc ion battery. *Appl Surf Sci* 2023; **638**: 158057.

60. Guo W, Cong Z, Guo Z et al. Dendrite-free Zn anode with dual channel 3D porous frameworks for rechargeable Zn batteries. *Energy Environ Sci* 2020; **30**: 104-112.

61. Wu B, Guo B, Chen Y et al. High Zinc Utilization Aqueous Zinc Ion Batteries Enabled by 3D Printed Graphene Arrays. *Energy Storage Mater* 2023; **54**: 75-84.
